# The Genome of *Borrelia recurrentis*, the Agent of Deadly Louse-Borne Relapsing Fever, Is a Degraded Subset of Tick-Borne *Borrelia duttonii*

**DOI:** 10.1371/journal.pgen.1000185

**Published:** 2008-09-12

**Authors:** Magali Lescot, Stéphane Audic, Catherine Robert, Thi Tien Nguyen, Guillaume Blanc, Sally J. Cutler, Patrick Wincker, Arnaud Couloux, Jean-Michel Claverie, Didier Raoult, Michel Drancourt

**Affiliations:** 1 Structural and Genomic Information Laboratory, CNRS UPR2589, IFR88, Parc Scientifique de Luminy, Marseille, France; 2 Unité des Rickettsies, UMR CNRS-IRD 6236, IFR48, Faculté de Médecine, Université de la Méditerranée, Marseille, France; 3 School of Health and Bioscience, University of East London, Stratford, London, United Kingdom; 4 Genoscope (CEA), Evry, France; Progentech, United States of America

## Abstract

In an effort to understand how a tick-borne pathogen adapts to the body louse, we sequenced and compared the genomes of the recurrent fever agents *Borrelia recurrentis* and *B. duttonii*. The 1,242,163–1,574,910-bp fragmented genomes of *B. recurrentis* and *B. duttonii* contain a unique 23-kb linear plasmid. This linear plasmid exhibits a large polyT track within the promoter region of an intact variable large protein gene and a telomere resolvase that is unique to *Borrelia*. The genome content is characterized by several repeat families, including antigenic lipoproteins. *B. recurrentis* exhibited a 20.4% genome size reduction and appeared to be a strain of *B. duttonii*, with a decaying genome, possibly due to the accumulation of genomic errors induced by the loss of *rec*A and *mut*S. Accompanying this were increases in the number of impaired genes and a reduction in coding capacity, including surface-exposed lipoproteins and putative virulence factors. Analysis of the reconstructed ancestral sequence compared to *B. duttonii* and *B. recurrentis* was consistent with the accelerated evolution observed in *B. recurrentis*. Vector specialization of louse-borne pathogens responsible for major epidemics was associated with rapid genome reduction. The correlation between gene loss and increased virulence of *B. recurrentis* parallels that of *Rickettsia prowazekii*, with both species being genomic subsets of less-virulent strains.

## Author Summary

Borreliae are vector-borne spirochetes that are responsible for Lyme disease and recurrent fevers. We completed the genome sequences of the tick-borne *Borrelia duttonii* and the louse-borne *B. recurrentis*. The former of these is responsible for emerging infections that mimic malaria in Africa and in travellers, and the latter is responsible for severe recurrent fever in poor African populations. Diagnostic tools for these pathogens remain poor with regard to sensitivity and specificity due, in part, to the lack of genomic sequences. In this study, we show that the genomic content of *B. recurrentis* is a subset of that of *B. duttonii*, the genes of which are undergoing a decay process. These phenomena are common to all louse-borne pathogens compared to their tick-borne counterparts. In *B. recurrentis*, this process may be due to the inactivation of genes encoding DNA repair mechanisms, implying the accumulation of errors in the genome. The increased virulence of *B. recurrentis* could not be traced back to specific virulence factors, illustrating the lack of correlation between the virulence of a pathogen and so-called virulence genes. Knowledge of these genomes will allow for the development of new molecular tools that provide a more-accurate, sensitive, and specific diagnosis of these emerging infections.

## Introduction

Spirochetes of the genus *Borrelia* are bacterial pathogens responsible for relapsing fever and Lyme borreliosis. Whereas the Lyme disease agents *Borrelia burgdorferi* [1],[2], *Borrelia garinii* [3], and *Borrelia afzelii* [4] are transmitted by hard ticks, the numerous relapsing fever borreliae are typically transmitted by soft ticks. Interestingly, tick-borne relapsing fever borreliae, including *Borrelia duttonii*, have shown extended vectorial capacity, whereas transmission of *Borrelia recurrentis*, which causes louse-borne relapsing fever, is restricted to *Pediculus humanus* [5],[6]. Besides their mode of transmission, these two highly related species of *Borrelia* exhibit very different epidemiological and clinical features. *B. duttonii* is endemic in Western Africa, where it demonstrates the highest incidence among all bacterial infections and causes up to six relapses, no mortality, and adverse perinatal outcomes [7]. In contrast, *B. recurrentis*, once responsible for worldwide outbreaks, is currently limited to Ethiopia and its surrounding countries [8]. It causes fewer relapses, but spontaneous mortality remains as high as 2–4% despite antibiotics, with patients suffering from distinctive hemorrhagic syndrome [9]. In addition, women who develop relapsing fever during pregnancy have a high incidence of spontaneous abortion [10]. Indeed, *B. recurrentis* and other louse-borne pathogens, including the typhus agent *Rickettsia prowazekii* [11] and the trench fever agent *Bartonella quintana* [12], exhibit higher virulence than their respective tick-borne relatives *B. duttonii*, *Rickettsia conorii* [13], and *Bartonella henselae* [12].

Borreliae are unique among bacteria in that their genome is comprised of a linear chromosome and both linear and circular plasmids [14]. We sequenced the genomes of *B. duttonii* and *B. recurrentis* to gain new insights into the structure and evolution of the borreliae.

## Results

### Genome Organization of *B. duttonii* and *B. recurrentis*

**Figure 1:**
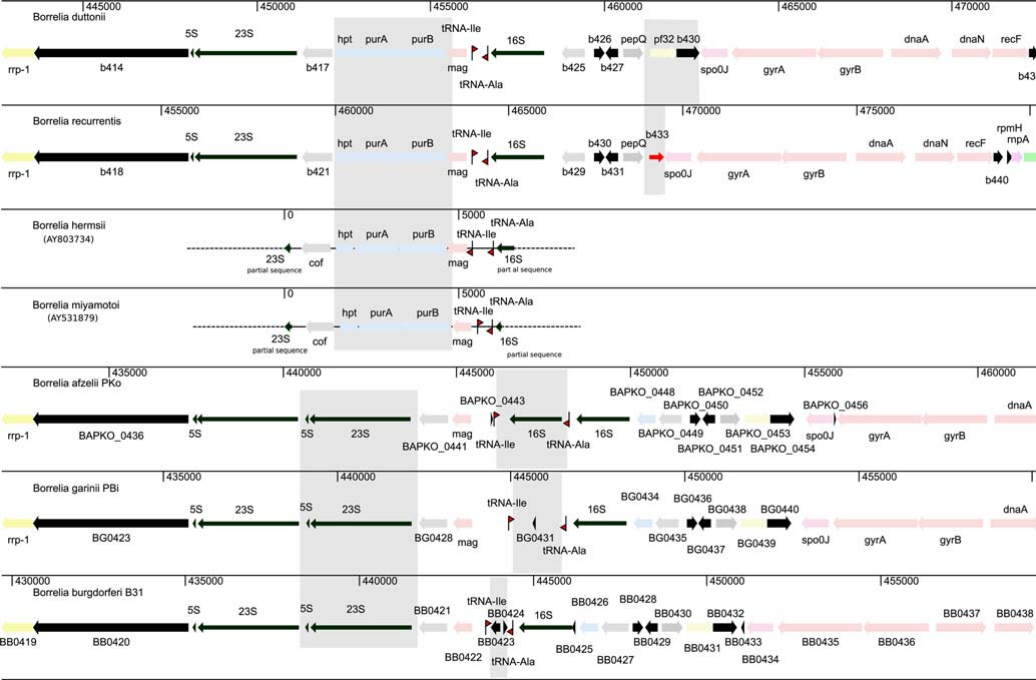
Genomic region around the chromosomal origin of replication in *B. recurrentis*, *B. duttonii*, *B. hermsii*, *B. miyamotoi*, *B. burgdorferi*, *B. garinii*, and *B. afzelii*. Insertion of *hpt*, *pur*A, and *pur*B is specific to the recurrent fever group borreliae. Duplication of 5S–23S rDNA is specific to the Lyme disease group borreliae. Variable spacing was observed between the Ala and Ile tRNAs. Specific degradation in the 5′ genomic region of *spo0*J was observed in *B. recurrentis*. Genes are colored according to their predicted functional category (Figure S1). Shaded areas correspond to regions of difference.

**Figure 2:**
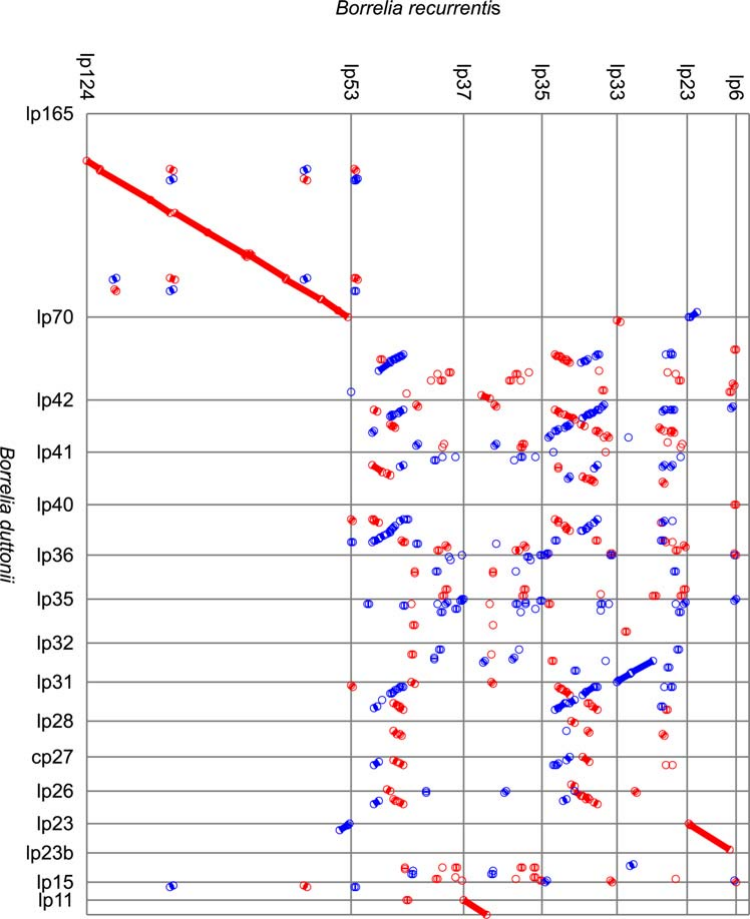
Dot plot showing the extensive similarity between *B. recurrentis* and *B. duttonii* plasmids. This figure was constructed using the NUCmer program from the MUMmer package. Red segments correspond to same strand matches, while blue segments correspond to opposite strand matches.

**Figure 3:**
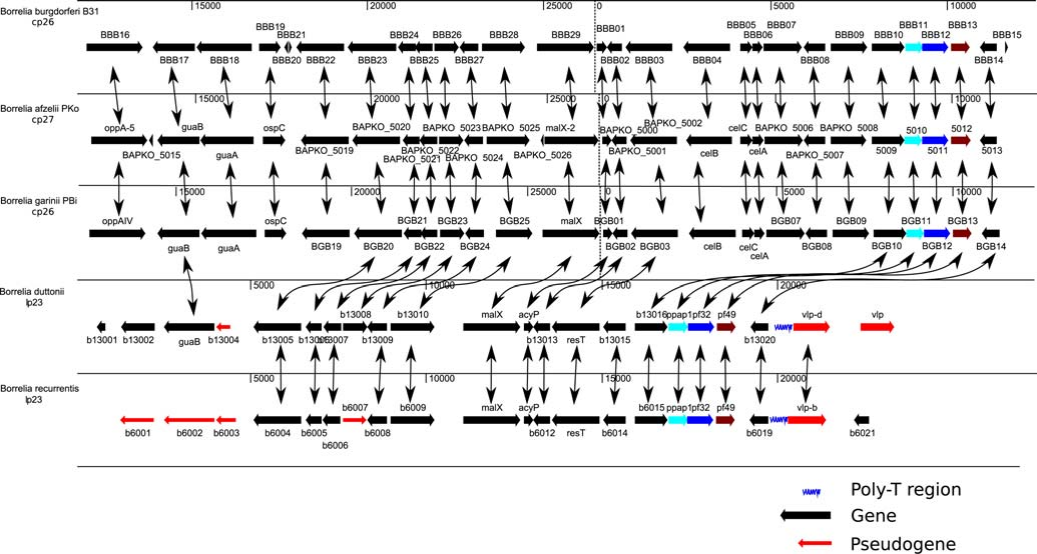
Comparison between recurrent fever group lp23 linear plasmid and *B. burgdorferi*/*B. garinii*-cp26, and *B. afzelii*-cp27 encoding telomere resolvase indicates a common structure. The large poly-T track in the promoter region of an intact vlp gene was specific to recurrent fever borreliae.

While the 1,242,163 bp *B. recurrentis* A1 strain genome contains only 8 linear fragments of 930,981-6,131 bp, the 1,575,296 bp *B. duttonii* Ly strain genome contains 17 linear fragments of 931,674-11,226 bp and one 27,476 bp circular fragment (Table 1, Figures S1 and S2, Genbank accession numbers CP000976-CP000992 for *B. duttonii* and CP000993-CP001000 for *B. recurrentis*). For each species, we designated the largest fragment as the chromosome and the smaller ones as the plasmids. The organization of the chromosome was conserved among borreliae, with *spo*OJ, *gyr*A, *gyr*B, *dna*A, and *dna*N (BDU_431-435, BRE_434-438) being clustered around the putative origin of replication near the GC/AT skew cross point (Figure 1 and Figure S3). In both species, the sole rrs operon (BDU_415-416, BDU_424, BRE_419-420, BRE_428), which is close to the putative origin of replication, was split by *hpt*, *pur*A, and *pur*B (BDU_418-420, BRE_422-424), as reported for *B. hermsii* and other relapsing fever borreliae [15],[16] (Figure 1). We also found similarity between the *B. duttonii*-circular plasmid (cp) 27, *B. duttonii*-linear plasmid (lp) 26, and *B. duttonii*-lp28. In addition, colinearity was observed between *B. duttonii*-lp23/*B. recurrentis*-lp23, *B. duttonii*-lp11/*B. recurrentis*-lp37, *B. duttonii*-lp32/*B. recurrentis*-lp33, *B. duttonii*-lp(26,28,31,40–42,70)/*B. recurrentis*-lp(35,53), and *B. duttonii*-lp165/*B. recurrentis*-lp124 (Figure 2). The latter plasmid has no counterpart among Lyme group borreliae. In both species, the linear plasmid lp23, which is syntenic to the circular plasmids *B. burgdorferi*/*B. garinii*-cp26 and *B. afzelii*-cp27, was particularly interesting. This plasmid exhibited a large polyT track (174 nucleotides in *B. duttonii* and 46 in *B. recurrentis*) of a length not previously reported in other bacteria, although T-rich regions containing Ts in 16 of 20 positions and Ts in 18 of 20 positions have been reported in the ospAB and vmp promoters of *B. burgdorferi* and *B. hermsii*, respectively [17]. This polyT track is located in the promoter of an intact variable large protein (*vlp*, BDU_13021, BRE_6020) gene situated at the telomere (Figure 3). This locus has been shown to be the site of *vlp* expression in *B. recurrentis* [18]. Strikingly, this plasmid encodes the unique telomere resolvase (*res*T, BDU_13014, BRE_6013), a protein specific to *Borrelia* species (Figure 3) [19],[20]. In *B. duttonii* and *B. recurrentis*, lp23 lacks the *cel*ABC genes involved in the PTS cellobiose system as well as *opp*A compared to other *Borrelia*.

**Table 1:** General features of the *Borrelia* genomes. Size is given in base pair (bp).

	*B. burgdorferi* *	*B. garinii* *	*B. afzelii* *	*B. duttonii*	*B. recurrentis*
**Genome size**	1,519,132	986,914	1,201,674	1,574,910	1,242,163
**Chromosome size**	910,725	904,246	905,394	931,674	930,981
**Plasmid size**	609,132	82,668	296,280	643,236	311,182
**Chromosome GC %**	28.6	28.3	28.3	27.6	27.5
**Plasmid number** (Linear)	21 (12)	2 (1)	8 (6)	16 (15)	7 (7)
**Total ORF number**	1,640	932	1,215	1,308	990
**Chromosome ORF number** (pseudogene)	851(7)	832 (8)	856 (6)	820 (2)	800 (20)
**Ribosomal RNAs**	6	5	5	3	3
**Transfert RNAs**	18	31	33	32	32

*: data from GenBank.

### Comparative Chromosomal Gene Content (Table S1)

Aside from the variable number of copies of repeated genes (see below), a few genomic differences were found between *B. duttonii* and *B. recurrentis*. There was a difference in the number of the protein genes encoded by the chromosome (820 genes in *B. duttonii* and 800 in *B. recurrentis*). Five genes (*rec*J, putative membrane protein, *rps*U, *fts*K, *bac*A, BDU_257-262, BRE_256-260, BRE_261-265) were duplicated in *B. recurrentis*, with one copy of *rec*J (BRE_261) presenting a frameshift and one copy of *bac*A (BRE_260) containing a frameshift and partial deletion. Four genes, pantothenate permease (*panF*, BDU_821/826, BRE_824), pseudouridylate synthase (*rluA*, BDU_822/827, BRE_825), an uncharacterized conserved protein (BDU_823/828, BRE_826), and UDP-N-acetylmuramate-alanine ligase (*murC*, BDU_824/829, BRE_827), were duplicated in *B. duttonii*. An ATPase involved in chromosome partitioning (homolog to Soj, BDU_429), close to the replication origin was lacking in *B. recurrentis*.

An in-frame STOP codon (tga, replacing tgg in *B. duttonii*) was found in the *B. recurrentis* copy of *rec*A (BDU_135, BRE_134), involved in the RecBCD dsDNA end repair pathway and the RecFOR ssDNA gap repair pathways [21] (Table S2A). We also found that *mutS* (BDU_101,BRE_100) and *smf* (BDU_300,BRE_304), genes belonging to the DNA processing DpRA family that collaborates with *recA* for recombination and bacterial transformation, were both impaired in *B. recurrentis* [22], with an in-frame STOP codon in *smf* (taa replacing caa) and a frameshift in *mut*S. Other impaired genes were found in *B. recurrentis* that are implicated in the following processes: maltose transport and metabolism (*mal*X , BDU_119, BRE_118 and *mal*Q, BDU_165, BRE_164, frameshifts), glycerol metabolism (*glp*A, BDU_244, BRE_243 and *glp*K, BDU_241, BRE_240, frameshifts), and adaptation to host environments (*opp*A1 transporter, BDU_329, BRE_333, internal STOP codon taa replacing caa). Other disrupted genes in *B.* recurrentis were *ypl*Q (BDU_120, BRE_119, frameshift), encoding a hemolysin III, *xyl*R2 (BDU_843, BRE_841, frameshift) of the xylose operon, the A subunit of an ATP-dependant Clp protease (BDU_364, BRE_368, frameshift), and an uncharacterized conserved protein (BDU_743, BRE_746, frameshift). Finally, a p35-like antigen (BDU_1), similar to the *B. burgdorferi* fibronectin-binding lipoprotein BBK32, was absent in *B. recurrentis*.

### Gene Families in *B. duttonii* and *B. recurrentis*

A significant number of *Borrelia* genes corresponded to repeat families, including variable major proteins (Vmp) and *Borrelia* direct repeats (Bdr). Most of these were plasmid-borne paralogous families [2]. To further study this phenomenon and compare different *Borrelia* species, we grouped together all predicted protein coding genes of *B. duttonii*, *B. recurrentis*, *B. burgdorferi*, *B. garinii*, and *B. afzelii* (see Materials and Methods). This analysis indicated that the most abundant families were those of the variable major proteins (vmp, including 600-bp vsp and 1000-bp vlp) [23], *Borrelia* direct repeats (*Bdr*), and plasmid partition proteins PF32, PF49, ppap1, and ppap2 (Table 2).

**Table 2:** *Borrelia* gene families.

Family number	Bdu	Bre	Bbu	Bga	Baf	Annotation
1	68	17	0	0	0	Vlp
5	18	9	18	0	7	bdr Borrelia direct repeat
2	16	9	21	3	12	pf32 plasmid partition protein
10	14	10	1	1	1	vsp genes (OspC)
3	15	7	22	2	10	pf49 plasmid partition protein
4	14	8	21	2	9	ppap1 plasmid partitionning associated protein 1
13	12	9	0	0	0	Uncharacterized conserved protein (lipoprotein)
44	14	0	0	0	0	Uncharacterized conserved protein (lipoprotein)
6	10	2	22	1	9	ppap2 plasmid partition associated protein 2 /ORFe-like protein
8	6	6	7	6	6	ABC-transport system, ATB-binding protein
14	8	2	8	0	1	family 115-like protein
54	8	2	0	0	0	ORFk-like protein
20	6	3	7	0	2	Mlpl-like lipoprotein
55	8	1	0	0	1	antigen P35-like protein
7	9	0	24	2	4	Uncharacterized conserved protein
76	2	6	0	0	1	Uncharacterized conserved protein
84	3	5	0	0	0	putative lipoprotein
9	4	4	4	5	10	Basic membrane protein BmpABCD
33	4	3	3	3	3	PTS system component
70	6	1	1	1	1	single-stranded DNA-binding protein
15	5	1	9	2	2	bsr gene
21	6	0	9	1	2	BppB
24	5	1	9	1	2	Uncharacterized conserved protein
31	5	1	8	1	2	Uncharacterized conserved protein
37	3	3	3	3	3	ABC transporter, ATP-binding protein

Family number refers to Dataset S1. The number of occurrences of genes of each family are reported for each of the *B. duttonii* (Bdu), *B. recurrentis* (Bre), *B. burgdorferi* (Bbu), *B. garinii* (Bga), *B. afzelii* (Baf), genomes. The table is sorted according to the number of member in *B. duttonii* and *B. recurrentis*.

Most Vmps are encoded by linear plasmids, and only two and three copies were found at the beginning of the *B. recurrentis* and *B. duttonii* chromosome, respectively (Table S3). The *vlp* family genes, similar to VlsE in Lyme disease borreliae, encode lipoproteins that, as a result of antigenic variation, allow relapsing fever borreliae to escape the host immune response [24]. *B. duttonii* encodes 68 *vlp* copies (19 with the consensus GGAGG of Ribosomal Binding Site), while *B. recurrentis* encodes 17 *vlp* copies (6 with the consensus GGAGG of Ribosomal Binding Site) (Table S3, Figure S4). Phylogeny clearly indicated that *vlps are* grouped into 4 subfamilies designated α, β, γ, and δ (Figure S4), as previously found for *B. hermsii* [23]. The largest subfamily is γ, with 26 *vlp* copies in *B. duttonii* and 9 in *B. recurrentis*. While numerous *vlp* pseudogenes were found in both genomes, *B. recurrentis* showed a tendency to lose intact *vlps*, with one *vlp* every 18-kb (on average, excluding the chromosome) compared with one *vlp* every 9.5-kb for *B. duttonii*. We identified remnants of 46 vlp genes in *B.duttonii* and 29 in *B. recurrentis*. The *vsp* family genes are related to the lipoprotein ospC present in Lyme disease borreliae. We identified 14 vsp in *B. duttonii* and 10 in *B. recurrentis*. The ratio of intact *vlp* to *vsp* was 17/10 (1.7) in *B. recurrentis* and 68/14 (4.9) in *B. duttonii*.

The Bdr family is common to relapsing fever and Lyme disease group borreliae [25]. In *B. burgdorferi*, Bdr are characterized by temperature-independent, low expression level, inner membrane-localized immunogenic proteins that are organized into 6 families (A to F). Bdr genes are found on most plasmids, except for the large *B. duttonii*-lp165/*B. recurrentis*-lp124 plasmid, which was also devoid of *vlp* and *vsp*.

In *B. duttonii*, putative replication and partition genes were identified on most plasmids, and were usually organized as a set of the four consecutive genes: PF32, PF49, ppap1, ppap2 (ORFe in *B. burgdorferi*) [2]. In *B. recurrentis*, this organization was still apparent despite gene decay.

The Bmp family contains basic membrane protein genes encoding lipoproteins. These proteins are expressed in infected patients, and result from different gene rearrangements in the five borreliae (Figure S5). For instance, the protein BmpB-1 is present only in Lyme group borreliae and could thus be used as a Lyme-specific diagnostic test.

An abundant repeat family (Family 44, 14 members, Table 2) was found in *B. duttonii*, but not in *B. recurrentis*. Indeed, members of this family are located at the 5′-end of the *B. duttonii*-lp164 plasmid, a region that lacks a counterpart in *B. recurrentis*. It contains uncharacterized conserved lipoproteins that are predicted to represent 7.6% of the lipoproteins in *B. duttonii*.

### Comparison with the Lyme Disease Group *Borrelia*

Genome sequencing of *B. recurrentis* and *B. duttonii* provides the opportunity to compare the gene content between relapsing fever and Lyme disease group borreliae. Whole chromosome comparison (Figure S1) shows extensive conservation of gene content and gene order. In both groups, we found an intact RecBCD system, which is important for repairing double-stranded DNA ends, but a deficient RecFOR pathway. RecF and RecR proteins are associated with RecO in the reparation of single-stranded DNA; however, RecO is absent in all borreliae, potentially leading to deficient repair of single-stranded nicks. We observed only 13 genes specific to the Lyme disease group and 17 genes specific to the relapsing fever group (excluding bmp genes, Table S2B) in the chromosomes of borreliae.

As previously observed in *B. hermsii* [15],[16], chromosome-encoded genes involved in purine metabolism and salvage were similarly found in these relapsing fever borreliae, including adenylosuccinate synthase (*purA*, BDU_419, BRE_423), adenylosuccinate lyase (*purB*, BDU_420, BRE_424), and hypoxanthine phosphoribosyltransferase (*hpt*, BDU_422, BRE_425). They were located between the 16S and 23S ribosomal DNA. Other genes unique to the relapsing fever group borreliae included a putative adenine-specific DNA methyltransferase (BDU_467, BRE_470), a copper homeostasis protein (*cutC*, BDU_844, BRE_842), the sugar specific PTS family protein (*nagE*, BDU_838,BRE_836), a trypsin-like serine protease (BDU_797, BRE_800), an ATP-dependent helicase belonging to the DinG family (BDU_740, BRE_743), a TPR domain containing protein (BDU_737, BRE_740), a protein with similarity to a response regulator receiver (CheY) modulated serine phosphatase (BDU_523, BRE_526), *glpQ* (BDU_243, BRE_242), *glp*T (BDU_241, BRE_240), *ma*f protein (BDU_127, BRE_126), *hsp*20 heat shock protein (BDU_444, BRE_447), purine salvage pathway genes including peptidyl-prolyl cis-trans isomerase (BDU_407, BRE_411), and the *rec* family members RecN (BDU_313, BRE_317), RecF (BDU_436, BRE_439), and RecR (BDU_465, BRE_468). Likewise, *arcC* (Carbamate kinase, BDU_857, BRE_855), which is involved in glutamate, arginine and proline biosynthesis are specific to relapsing fever borreliae, but was impaired in *B. recurrentis*. Among these genes, 16 exhibited best homologs with sequences outside of the spirochetes group. Interestingly, 5 demonstrated good homology with *Fusobacterium nucleatum*, as described for another spirochete, *Treponema denticola* [26].

Conversely, some genes were only found on the Lyme disease group (Table S2B), including a putative L-sorbosone dehydrogenase, two antigens S2, an oligopeptide ABC transporter (*oppA-3*), a methylglyoxal synthase, a lipoprotein LA7, a basic membrane protein B (*bmpB-1*), an inositol monophosphatase, an aldose reductase, a MATE efflux family protein, a *pfs* protein (*pfs-2*), a *rep* helicase, a small primase-like protein, and an Na+/H+ antiporter (*nhaC-1*).

In contrast to what was observed for the chromosome, the plasmid contents of the relapsing fever group were very different from that of the Lyme disease group. Only three *B. duttonii* plasmids (lp165, lp70 and lp23) exhibited significant synteny with *B. burgdorferi* plasmids (Figure S6). *B. duttonii*-lp165 and *B. recurrentis*-lp124 encoded *nrd*F (ribonucleoside-diphosphate reductase beta subunit, BDU_1075, BRE_1045), *nrd*E (ribonucleoside-diphosphate reductase alpha subunit, BDU_1076, BRE_1046), and *nrd*I (auxiliary protein, BDU_1077, BRE_1047) (Table S2B), all of which were previously reported in *B. hermsii* [27], but were absent in the Lyme disease group of *Borrelia*. Using the SpLip program [28] with the *B. burgdorferi* matrix supplied by the authors, we retrieved 171 probable and 13 possible lipoproteins in *B. duttonii*, 80 (11) in *B. recurrentis*, 111 (9) in *B. burgdorferi*, 45 (8) in *B. garinii*, and 84 (10) in *B. afzelii*. Relapsing fever borreliae proteomes contain a larger fraction of lipoprotein (13.63% in *B. duttonii* and 8.72% in *B. recurrentis*) than Lyme disease group borreliae (7.74% in *B. afzelii*, 7.32% in *B. burgdorferi* and 5.9% in *B. garinii*).

### *Borrelia* Evolution

**Figure 4:**
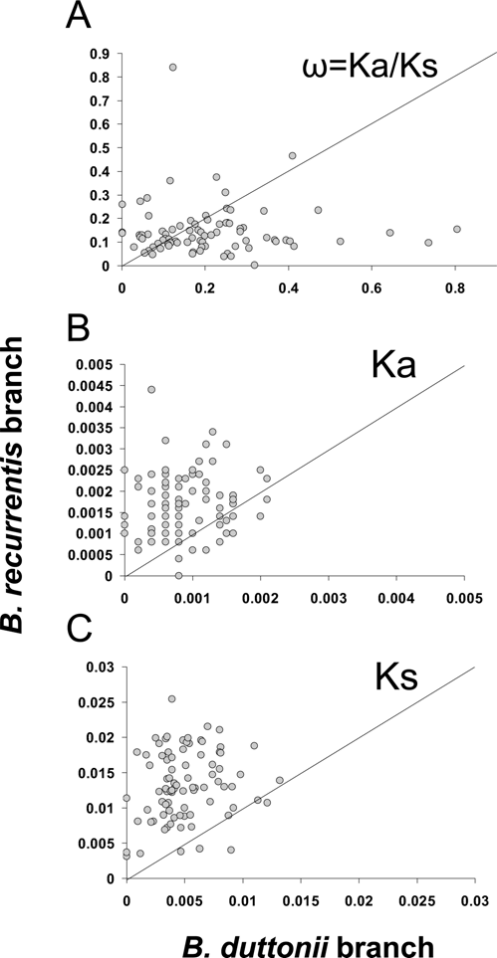
The ω = Ka/Ks, Ka, and Ks values for *B. recurrentis* and *B. duttonii* branches. Seventy-seven 2190-codon alignments derived from the initial concatenated alignment of the borrelia core set were analyzed using model 2. Only values obtained for the *B. recurrentis* and *B. duttonii* branches are presented. The dot plots show ω = Ka/ks (A), Ka (B), and Ks (C) values.

*B. duttonii* contained no impaired genes in its chromosome (except for two vlp pseudogenes), whereas *B. recurrentis* exhibits 20 impaired genes (Table S2A). This suggests that *B. recurrentis* evolved under more relaxed constraints (e.g. accumulated more deleterious mutations) than *B. duttonii*. This hypothesis was examined by analyzing the ratio of non-synonymous (Ka) to synonymous (Ks) substitution rates (denoted ω = Ka/Ks) among 773 conserved genes of the five borreliae. Based on the most suitable model of evolution (See Materials and Methods), the estimated ω ratio was nearly twice as high for the *B. recurrentis* branch (ω*_Bre_* = 0.18) than for the *B duttonii* branch (ω*_Bdu_* = 0.10). These results suggest that, on average, the genome of *B. recurrentis* tends to evolve under weaker coding sequence constraints than the genome of *B. duttonii*. In addition, the number of non-synonymous substitutions was higher in the *B. recurrentis* branch (n = 695) than in the *B. duttonii* branch (n = 366). This indicates that *B. recurrentis* proteins tend to diverge faster. To find out whether this acceleration was restricted to a specific subset of genes, we further analyzed sub-alignments comprising, on average, 10 genes. This analysis showed that ω*_Bre_* calculated for the sub-alignments were not systematically higher than ω*_Bdu_* (Figure 4A). This suggests that the selective constraints acting on coding sequences are, in general, not less effective in *B. recurrentis* than in *B. duttonii*. In contrast, the Ka and Ks values were almost systematically higher for *B. recurrentis* (Figure 4B and C). These results indicate that *B. recurrentis* genome is globally evolving faster that the one of *B. duttonii*.

## Discussion

### The Linear, Fragmented Genome of *Borrelia*

While circular chromosomes are most commonly seen in bacteria, linear chromosomes are encountered in some phylogenetically distinct species including *Agrobacterium tumefaciens* [29],[30], *Streptomyces* species [31],[32], and *Borrelia* species [1]–[4]. The latter are unique in that they harbor >3 linear genomic fragments, whereas the other sequenced spirochetes, *Treponema* [33],[26] and *Leptospira* [34]–[36], possess 1–2 circular chromosomes. This suggests that genome linearization is a recent evolutionary event in the spirochete lineage. Genome linearization of *Borrelia* is sustained by telomeres, terminal small inverted repeats with covalently closed hairpin ends [37],[38]. Similar features have been described for Poxvirus, African swine fever virus, Chlorella viruses, the mtDNA of yeasts and protozoa, and the *Escherichia coli* phage N15 [37]–[39]. Replication of telomeres from a bidirectional origin [40],[41] produces intermediates for which the replicated telomeres comprise dimer junctions between inverted repeats of the original plasmid [19]. Replicated telomeres are then processed by ResT, the essential *B. burgdorferi* cp26-encoded telomere resolvase responsible for a particular DNA breakage and reunion event that regenerates the hairpin telomeres [20],[42],[43]. When cp26 was deleted in *B. burgdorferi* cells, viability was lost [44]. ResT acts via a catalytic mechanism analogous to that of tyrosine recombinases and type IB topoisomerases [45]. We found ResT in relapsing fever *Borrelia*, in agreement with the concept of telomere-mediated genome linearization among these organisms. ResT was recently also shown to perform a reverse reaction that fuses telomeres from unrelated replicons. In the Lyme disease group, initiation of replication occurs in the central region of the linear chromosome that comprises a polar CG skew and proceeds bidirectionnaly [40],[46]. The observed parallel genome architecture suggests an identical replication mechanism among the relapsing fever group.

### *B. recurrentis*, a Decaying Strain of *B. duttonii*

Previous limited phylogenetic data based on 16S rDNA [6] and 16S–23S intergenic spacer [5] raised the question of whether *B. duttonii* and *B. recurrentis* are different species [47]. Gene content analysis showed that the genome of *B. recurrentis* is a subset of that of *B. duttonii*. The chromosomes of both species were found to be almost entirely colinear, and all *B. recurrentis* plasmids have a counterpart in *B. duttonii*. Altogether, 30 genes or gene families of *B. duttonii* were either absent, split, or reduced in number in *B. recurrentis*. In particular, a set of four consecutive genes, PF32, PF49, ppap1, and ppap2, involved in plasmid replication and partitioning were well conserved in most *B. duttonii* plasmids, but were damaged considerably in *B. recurrentis* plasmids. This suggests ongoing plasmid loss in *B. recurrentis*. Likewise, *B. recurrentis* lacks a chromosomal *Soj* homologue, which is involved in chromosome partitioning. Such reductive evolution may be linked to defective DNA repair in *B. recurrentis*. Indeed, the *B. recurrentis rec*A gene sequence presents an in-frame STOP codon. Although compensatory mechanisms that preserve the expression of *rec*A could not be ruled out, this finding was surprising, as *rec*A is a ubiquitous and highly conserved gene involved in DNA repair [21]. Impaired *rec*A was previously reported in *Spiroplasma melliferum* [48], whereas *Buchnera* and *Blochmania floridanus* lack this gene [49],[50]. In *Escherichia coli*, 50% of *rec*A mutants are viable and avoid chromosome lesions [51], but recA dut^*^ (dUTPase) mutants are lethal in the presence of *nfi*, which encodes endonuclease V (deoxyinosine 3′ endonuclease) [52]. Since *Borrelia* species lack *dut*, we hypothesize that the viability of *B. recurrentis* is maintained by the absence of *nfi*, as occurs in *B. burgdorferi*, *B. garinii*, and *B. duttonii*. We were unable to find either an ATP-dependant LigD or the DNA-end-binding-protein, Ku, involved in DNA repair by non-homologous end-joining [53]. The lack of an intact *recA* and *smf* in *B. recurrentis* may explain the observed accelerated evolution of its genome compared to *B. duttonii*. Taken together, the genomic data and phylogenetic data suggest that *B. recurrentis* is actually a strain of *B. duttonii*.

### Adaptation of Pathogens to the Body Louse Vector

Genome comparison of louse-borne bacteria with their tick-borne counterparts indicated an extensive genome size reduction of 20.4% for *Borrelia* spp., 18% for *Bartonella* spp., and 12.6% for *Rickettsia* spp. Among borreliae, genes that were lost included the antigenic lipoproteins *vlp* and *vsp*, genes involved in chromosome and plasmid partitioning, and genes involved in xylose and glycerate metabolism. Degradation of genes into pseudogenes within louse-borne species (128 *B. henselae* / 175 *B. quintana*; 2 *B. duttonii* / 20 *B. recurrentis*, Table S2A) suggests a progression toward the complete loss of these genes. Indeed, louse-borne species contain 21%–39% less CDSs than their tick-borne counterpart. This phenomenon is illustrated by the decreased number of repeat families from 43 in *B. henselae* to 11 in *B. quintana* [12], from 12 in *R. conorii* [13] to 3 in *R. prowazekii* [11], and from 54 in *B. duttonii* to 17 in *B. recurrentis*. Loss of DNA repair genes such as *mutM* and *mutT* in the typhus group *R. prowazekii* [54], and *rec*A, *mutS*, and *smf* in *B. recurrentis* may contribute to a higher rate of replication error, leading to faster genome decay among these louse-borne pathogens. Genomic differences between louse-borne species and their tick-borne counterparts may correlate with their concomitant adaptation to a human host [12]. A 4-nucleotide difference (0.26%) in the 16S rDNA sequence of *B. duttonii* and *B. recurrentis* estimates their divergence to have occurred between 6.5 and 13 million years ago [55]. This is roughly the same as the time of the divergence of the human specific louse vector of *B. recurrentis* and the common ancestral primate-associated ectoparasite [56]. We hypothesize that genome decay in louse-borne bacteria correlates with the host-specific bottleneck of the arthropod vector. Conversely, tick-transmitted organisms may adapt to diverse host populations, which is facilitated by tick feeding habits, unlike louse-borne pathogens. Such adaptation to body louse transmission is correlated with increased evolutionary rates illustrated in *B. recurrentis* analogous to those observed for *R. prowazekii* [54]. Genome size reduction and on-going gene and function decay in louse-borne pathogens illustrate the genomic fluidity associated with adaptation of bacteria from a large environmental niche to a more restricted one [57],[58].

### Antigenic Variability and Virulence Factors

Variation in the expression of a dominant surface antigen allows borreliae to evade immune defences. This evasion increases the duration and number of recurrences of bacteremia, and thus, the likelihood of subsequent transmission [14]. In *B. recurrentis* strain A1, Vlp has been shown to be the major pro-inflammatory molecule [59]. Furthermore, expression of certain lipoproteins, for instance in *Borrelia turicatae*, has been shown to modulate tissue tropism. Specifically, the Bt1 and Bt2 variants are predictive of either neurotropism or spirochetemia and arthritis, respectively [60],[61]. Detailed molecular analyses revealed that the corresponding genes are arranged into silent and expressed copies on different plasmids [62],[63]. Indeed, two copies of *vlp1_B. recurrentis A1_* were found in *B. recurrentis* [59]. This gene was identified as a pseudogene in lp53 and as an active gene in lp23 (lp23_20295_21386, BRE_6020). Antigenic variation occurs either by replacing the entire open reading frame of the expressed gene with a previously silent one, or by activating a previously silent downstream gene [64]. The likelihood of different antigenic variants being expressed appears not to be random, but is ordered in a semi-hierarchical fashion. This hierarchy depends on the sequence similarity between the upstream homology sequence located at the expression site of the variant gene and the distance separating the extragenic downstream homology sequence [65]. To date, the absence of suitable animal models has precluded antigenic variation studies among *B. recurrentis* and *B. duttonii*; however, the genome sequence data reported here could facilitate the molecular characterization of antigenic variants in clinical samples.

In contrast to Lyme disease spirochetes (<10^5^/ml), relapsing-fever spirochetes achieve high cell densities (>10^8^/ml) in patients' blood, suggesting differences in the ability of both groups to either exploit or survive in blood. It has been hypothesized that the purine salvage pathways are among these differences [16]. In particular, hypoxanthine, a primary product of purine catabolism, is exported to the outer surface of red blood cells. This could facilitate the direct uptake of hypoxanthine from red blood cells, providing a purine source for the synthesis of nucleotides by these borreliae [16]. In addition, some researchers have suggested that differences in glycerol-3-phosphate (G3P), an important metabolic intermediate for phospholipid synthesis, acquisition pathways contribute to differences in the density of borreliae in blood [66]. *B. recurrentis* has apparently inactivated *glp*A and *glp*K, indicating that two of the three G3P acquisition pathways in *Borrelia* have been turned-off in *B. recurrentis*. *B. recurrentis* could acquire G3P only by the hydrolysis of deacylated phospholipids from the erythrocyte membrane, in agreement with the fact that its body louse vector takes daily bloody meal in order to survive. Therefore, such a restriction would not be deleterious to *B. recurrentis*, but indeed exemplifies adaptation to a specific ecological niche [67]. As GlpQ is an immunodominant antigen used to discriminate between Lyme disease and relapsing fever groups [68], the present genomic data may help refine the serological diagnosis of relapsing fever group borrelioses.

Genome analysis revealed that *B. recurrentis* encodes fewer putative virulence factors than *B. duttonii*, an unexpected finding given the high mortality in untreated louse-borne relapsing fever [69]. In particular, *B. recurrentis* encodes a reduced proportion of major antigenic Vlp compared to Vsp lipoproteins than *B. duttonii*. It also lacks a hemolysin, which is present but is obviously degradated, as well as a p35-like antigen similar to the BBK32 fibronectin-binding lipoprotein of *B. burgdorferi*. Loss of intact *glp*A and *glp*K in *B. recurrentis* may limit the acquisition of glycerol-3-phosphate. It is also possible that the loss of one intact copy of *bac*A in *B. recurrentis* may cause increased virulence, as observed for *Brucella abortus*, in which *bac*A is deleted [70]. Other genes that are critical for the environmental survival of *B. recurrentis*, including the broad-spectrum peptide permease OppA-1 gene [71] and the ClpA chaperone, were also degraded. The ClpA chaperone prepares protein substrates for degradation by ClpP [72], a central complex that controls the stability and activity of transcriptional regulators during cell stress Impaired ClpA may deregulate transcription during *B. recurrentis* infection and lead to uncontrolled expression of virulence factors. Altogether, these defects may impair environmental sensing by *B. recurrentis*. These findings illustrate the lack of correlation between the observed virulence and the number of virulence factors possessed by an organism [73]. Finally, *B. recurrentis* illustrates the emerging concept that microbial virulence, for humans, may result from gene loss [58].

## Materials and Methods

### Isolation of Strains and Growth Conditions

*B. recurrentis* strain A1 isolated from an adult patient with louse-borne relapsing fever in Ethiopia [67] and *B. duttonii* strain Ly isolated from a 2-year-old girl with tick-borne relapsing fever in Tanzania [74] were grown on BSK-H complete medium batch number 057K4413 and 10K8402 (Sigma) at 37°C. Pulsed field gel electrophoresis (PFGE) was performed (CHEF-DRIII apparatus, Biorad) to determine the size of the genome and to analyze plasmid patterns under three different electrophoretic conditions. The samples were prepared as described previously [75]. Small plasmids could be visualized using a linear increase in pulse times between 1 to 3 sec. at 180 V over a 10 h period. Plasmids from 145 to 23 kb were detected using a linear increase in pulse time between 3 to 10 sec. at 180 V over a 15 h period, followed by an extensive migration using a linear increase in pulse time between 50 to 150 sec. at 180 V over a 30 h period (Figure S7).

### Shotgun Sequencing of *B. duttonii* and *B. recurrentis* Genomes and Sequencing Strategy

As attempts to isolate chromosome and plasmid DNA from PFEG after β-agarase treatment failed to produce sufficient DNA yield, genomic DNA was extracted from 25 ml of culture by incubation with 1% SDS-RNAseI (50 µg/ml) for 3 hours at 37°C, followed by proteinase K digestion (250 µg/ml) at 37°C overnight. After 3 phenol extractions, the DNA was precipitated with ethanol. The quality, yield, and DNA concentration were estimated by electrophoresis on agarose gels stained with ethidium bromide. Genomic DNA was sheared by mechanical fragmentation with a Hydroshear device (GeneMachines, San Carlos, California, USA) to construct plasmid libraries. After blunt end repair and *Bst*XI adapter ligation, fragments of 2 kb, 5 kb, and 10 kb were cloned into the high copy number vector pCDNA2.1 (Invitrogen, Life Technologies) digested with *Bst*XI. Transformations were performed using the electrocompetent *E. coli* strain DH10B (Invitrogen, Life Technologies). Each library was validated using 96 clones from which the insert size was estimated by agarose gel electrophoresis. Sequencing using vector-based primers was carried out using the ABI 3730 Applera sequencer. For *B. duttonii*, only libraries of 2 kb and 10 kb were sequenced, producing 14,719 and 10,066 reads, respectively. For *B. recurrentis*, three shotgun libraries of 2 kb, 5 kb, and 10 kb generated 14,794, 2,248, and 2,042 reads, respectively. Reads were analyzed and assembled into contigs using the Phred, Phrap, and Consed software packages [76]–[78]. Finishing was performed to verify low quality regions, to fill-in sequences by DNA walking using subcloned DNA, and to close gaps. A total of 1,034 *B. duttonii* specific primers and 784 *B. recurrentis* primers were designed. All finishing sequencing reactions were carried out on an ABI 3130 Applera sequencer.

### Annotation of *Borrelia recurrentis* and *Borrelia duttonii* Sequences

An initial set of protein-coding genes was detected using self-training Markov models [79] and careful examination of intergenic regions to rescue additional genes. Putative protein coding genes were then validated and annotated by sequence similarity using BlastP [80] against the non-redundant protein database from the National Center for Biotechnology Information (NCBI) and the KEGG protein database [81]. Putative protein coding genes were also validated by profile detection using RPSblast [80] and the COG database [82]. Genes encoding tRNA were identified with tRNAscan-SE [83], and other RNAs were located using BlastN [80]. Dot plots of plasmids from both species were computed using the NUCmer program from the MUMmer package [84].

### Gene Families

To compare the distribution of genes in different *Borrelia* families, we grouped together all predicted protein coding genes for *B. duttonii* (this work), *B. recurrentis* (this work), *B. burgdorferi* (GenBank: NC_000948-57, NC_001318, NC_001849-57, NC_001903, NC_001904), *B. garinii* (GenBank: NC_006128, NC_006129, NC_006156), and *B. afzelii* (GenBank: NC_008273, NC_008274, NC_008277, NC_008564-69), by performing a mutual BlastP comparison of this set of genes. The resulting comparison data were submitted to a Markov Chain Clustering algorithm to regroup the genes into families [85]. The resulting set of clustered sequences is available as Dataset S1. The same analysis was performed on the individual proteome of *B. henselae*, *B. quintana*, *R. prowazekii*, *R. conorii*, *B. duttonii*, and *B. recurrentis* to count the number of repeat families containing at least 3 members in each of these genomes.

### Lipoproteins

Lipoprotein computational prediction has been the subject of a specific article [28] that describes the SpLip program used in the present work.

### Analysis of *Borrelia* Evolution

The 856 proteins of the *B. burgdorferi* chromosome were aligned with the other *Borrelia* (*B. duttonii*, *B. recurrentis*, *B. garinii* and *B. afzelii*) proteomes using the BlastP program (e-value<1e-10) [80]. We identified 773 genes that were conserved in all borreliae (borreliae core genes) using the reciprocal best Blast hit criterion. The 773 *Borrelia* core proteins were first aligned individually using MUSCLE [86]. Poorly aligned regions were discarded by GBLOCKS [87]. The resulting alignments were used as a guide to align the corresponding coding sequences on a codon basis. After cleaning up the nucleotide alignments for poorly aligned regions, the 773 multiple alignments were concatenated in a single alignment of 169,249 codons. Estimation of the ω = Ka/Ks ratio was performed using the maximum likelihood method implemented in the CODEML program [88]. The ω ratio measures the magnitude and direction of selective pressure on coding sequence, with ω = 1, <1, and >1 indicating neutral evolution, purifying selection, and positive diversifying selection, respectively. To examine whether the ω ratio varied between the *B. recurrentis* and *B. duttonii* branches, we fitted two different models: the first model considered a single ω ratio for the 2 branches of *B. recurrentis* and *B. duttonii* (ω*_Bre-Bdu_*) and a background ω ratio (ω_0_) averaged over the remaining branches of the borrelia phylogeny. In the second model, a specific ω ratio was considered for each of the *B. recurrentis* and *B. duttonii* branches (ω*_Bre_* and ω*_Bdu_*, respectively) as well as a background ω_0_ ratio common to the remaining branches. To determine which of the two nested models best fit the data, we compared their likelihoods using the Likelihood Ratio Test (LRT)(Table S4). The likelihood statistics – i.e. twice the log likelihood difference between the 2 models (2δlnL), can be compared to the chi square distribution with a degree of freedom equal to the difference of the number of free parameters in the two models (ddf = 1 in our analysis). The LRT test (2δlnL = 6.0) indicated that model 2 better fits the data than model 1. However, the likelihood difference between the two models is only borderline significant (*P* = 0.014).

## Supporting Information

Figure S1 Whole chromosome display of sequenced borreliae, including the recurrent fever group *B. duttonii* and *B. recurrentis* and the Lyme disease group *B. burgdorferi*, *B. garinii*, and *B. afzelii*. Genes are colored according to their predicted functional category. Highlighted areas correspond to regions of difference.
(9.45 MB PDF)

Figure S2 *B. duttonii* and *B. recurrentis* plasmids. The large *B. duttonii*-lp165 and *B. recurrentis*-lp124 plasmids, which demonstrate extensive similarity, are shown side by side, with shaded areas indicating regions of difference. Genes are colored according to their repeat-family membership (Table 2).
(1.78 MB PDF)

Figure S3 GC and AT skews of *B. recurrentis* and *B. duttonii* chromosomes showing reversal near the origin of replication.
(0.05 MB PDF)

Figure S4 Phylogenetic tree of intact vlp genes in the genomes of *B. duttonii* (in red) and *B. recurrentis* (in blue). The genes were aligned with the MUSCLE program [86] and the tree was built using PHYML [89].
(0.40 MB PDF)

Figure S5 Comparison of the Bmp gene family in five borreliae genomes indicates structural rearrangements in Lyme disease group borreliae. Genes are colored according to predicted functional category (Figure S1).
(0.15 MB PDF)

Figure S6 Dot plot showing the extensive similarity between *B. duttonii* and *B. burgdorferi* plasmids. This figure was constructed using the PROmer program from the MUMmer package. Red segments correspond to same strand matches, while blue segments correspond to opposite strand matches.
(0.07 MB PDF)

Figure S7 Pulse field gel electrophoresis images of *B. duttonii* and *B. recurrentis*.
(0.15 MB PDF)

Table S1 List of genes which are either absent, split, or in reduced number in *B. recurrentis* when compared to *B. duttonii*.
(0.03 MB DOC)

Table S2 A. Split and truncated genes on the *Borrelia* chromosome. B. List of genes unconserved between the five borreliae.
(0.08 MB DOC)

Table S3 List of the different variable large proteins in the *B. duttonii* and *B. recurrentis* genomes. A. *B. recurrentis*; B. *B. duttonii*; C. Repartition of the Vlp genes among different classes in the two borreliae.
(0.15 MB DOC)

Table S4 Parameters of the codon models used in this study.
(0.02 MB DOC)

Dataset S1 List of the predicted proteins, in fasta format, of *B. duttonii*, *B. recurrentis*, *B. burgdorferi*, *B. garinii* and *B. afzelii* grouped in families.
(2.59 MB TXT)
