# Supplementary material for: The Genome of Borrelia recurrentis, the Agent of Deadly Louse-Borne Relapsing Fever, Is a Degraded Subset of Tick-Borne Borrelia duttonii
Source: PLoS Genet. 2008 Sep 12;4(9):e1000185. doi: 10.1371/journal.pgen.1000185 (PMC2525819; doi:10.1371/journal.pgen.1000185)
Supplement: Figure S1 — Whole chromosome display of sequenced borreliae, including the recurrent fever group B. duttonii and B. recurrentis and the Lyme disease group B. burgdorferi, B. garinii, and B. afzelii. Genes are colored according to their predicted functional category. Highlighted areas correspond to regions of difference. (9.45 MB PDF) [file pgen.1000185.s001.pdf]

Whole sequenced borreliae chromosome display including recurrent fever group *B. duttonii* and *B. recurrentis* and Lyme disease group *B. burgdorferi*, *B. garinii* and *B. afzelii*.

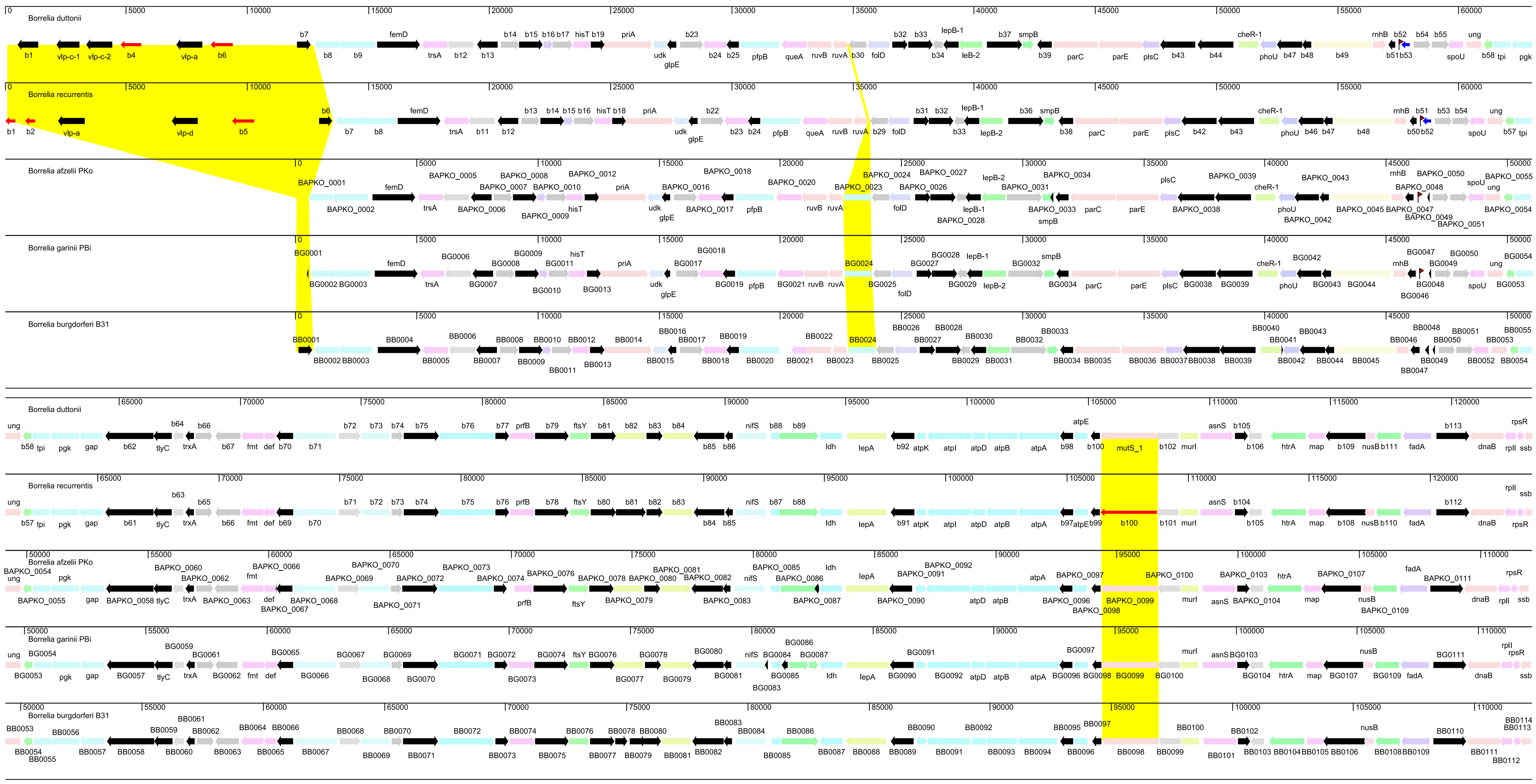

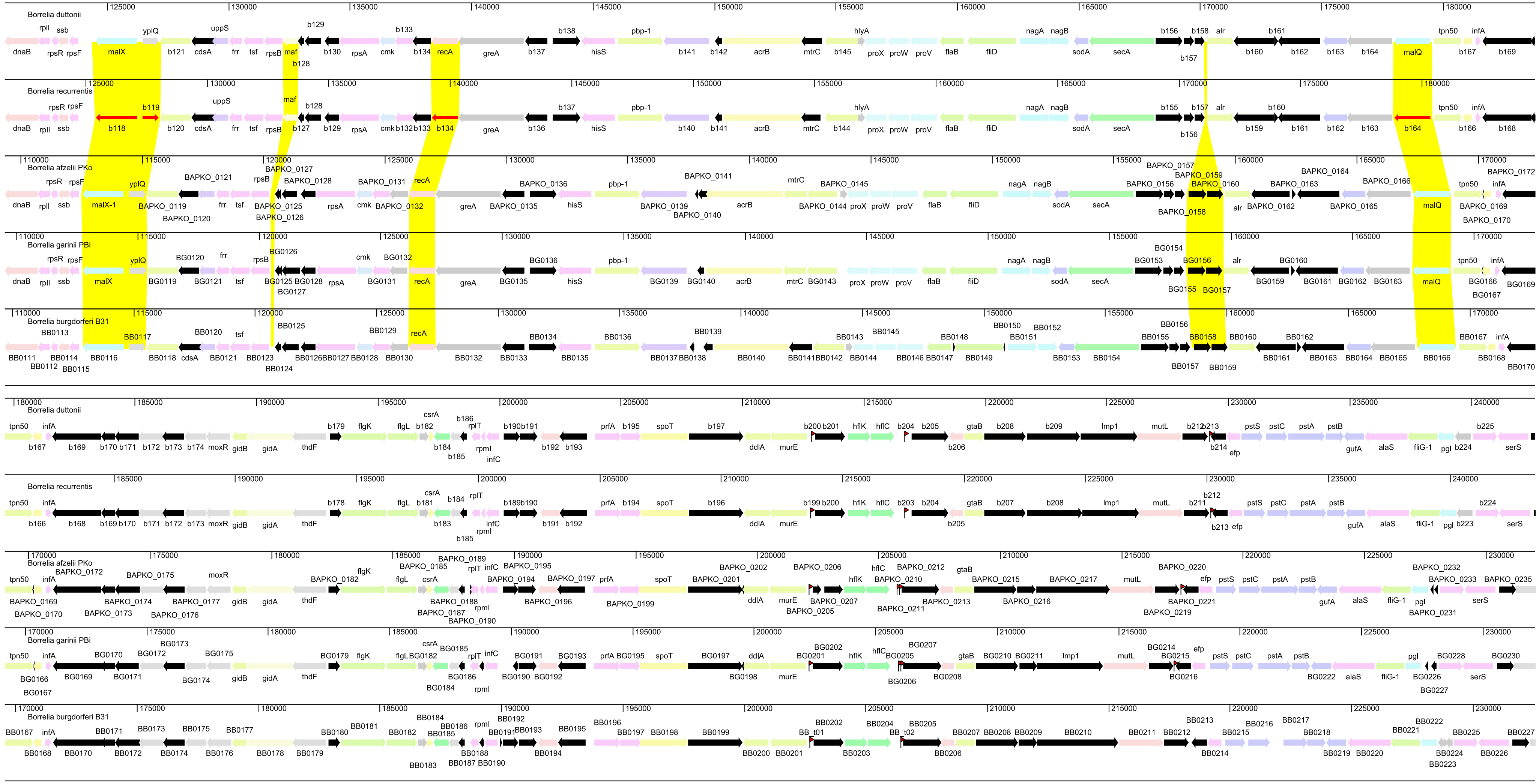

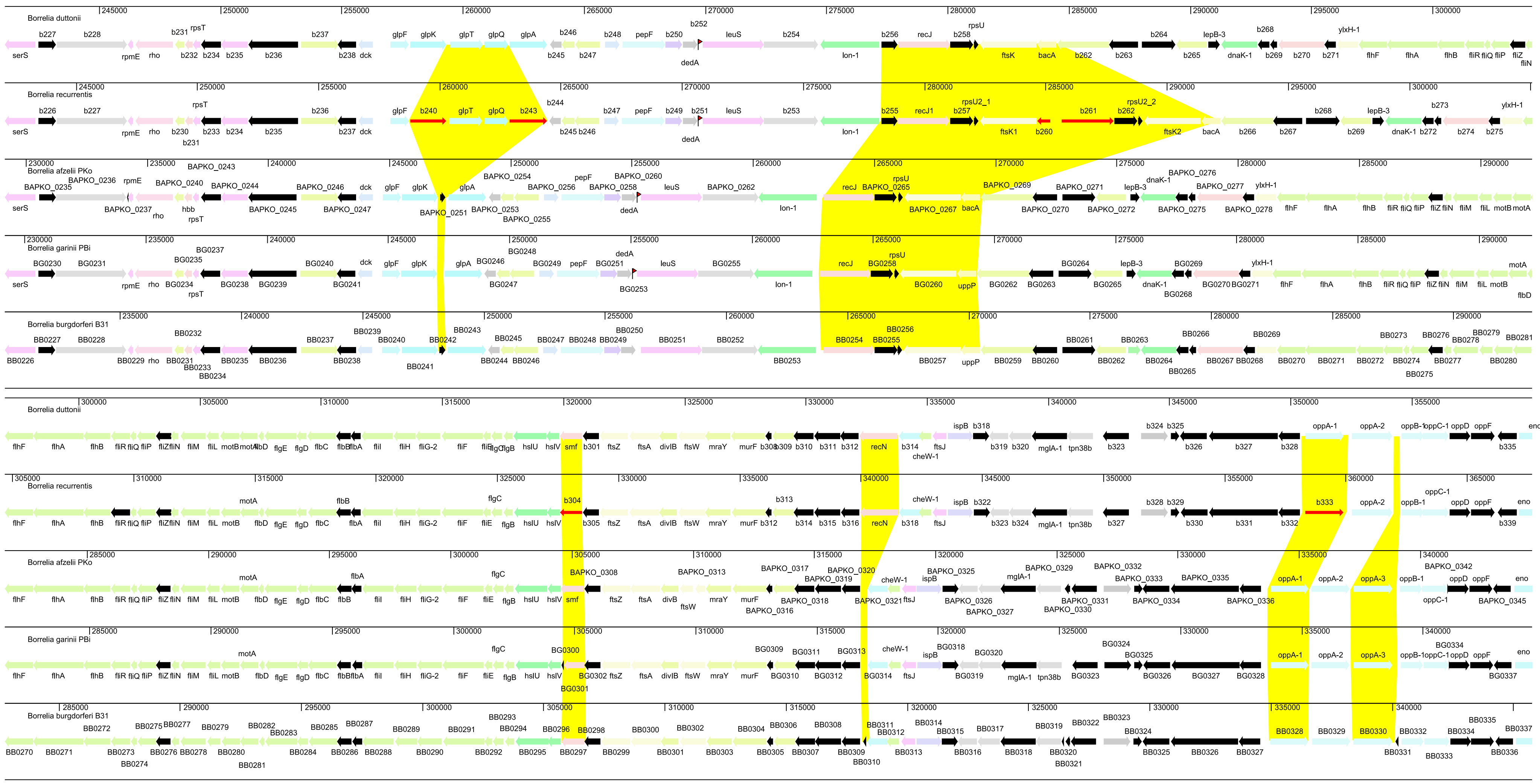
Borrelia duttonii
Borrelia recurrentis
Borrelia afzelii PKo
Borrelia garinii PBi
Borrelia burgdorferi B31
Borrelia duttonii
Borrelia recurrentis
Borrelia afzelii PKo
Borrelia garinii PBi
Borrelia burgdorferi B31

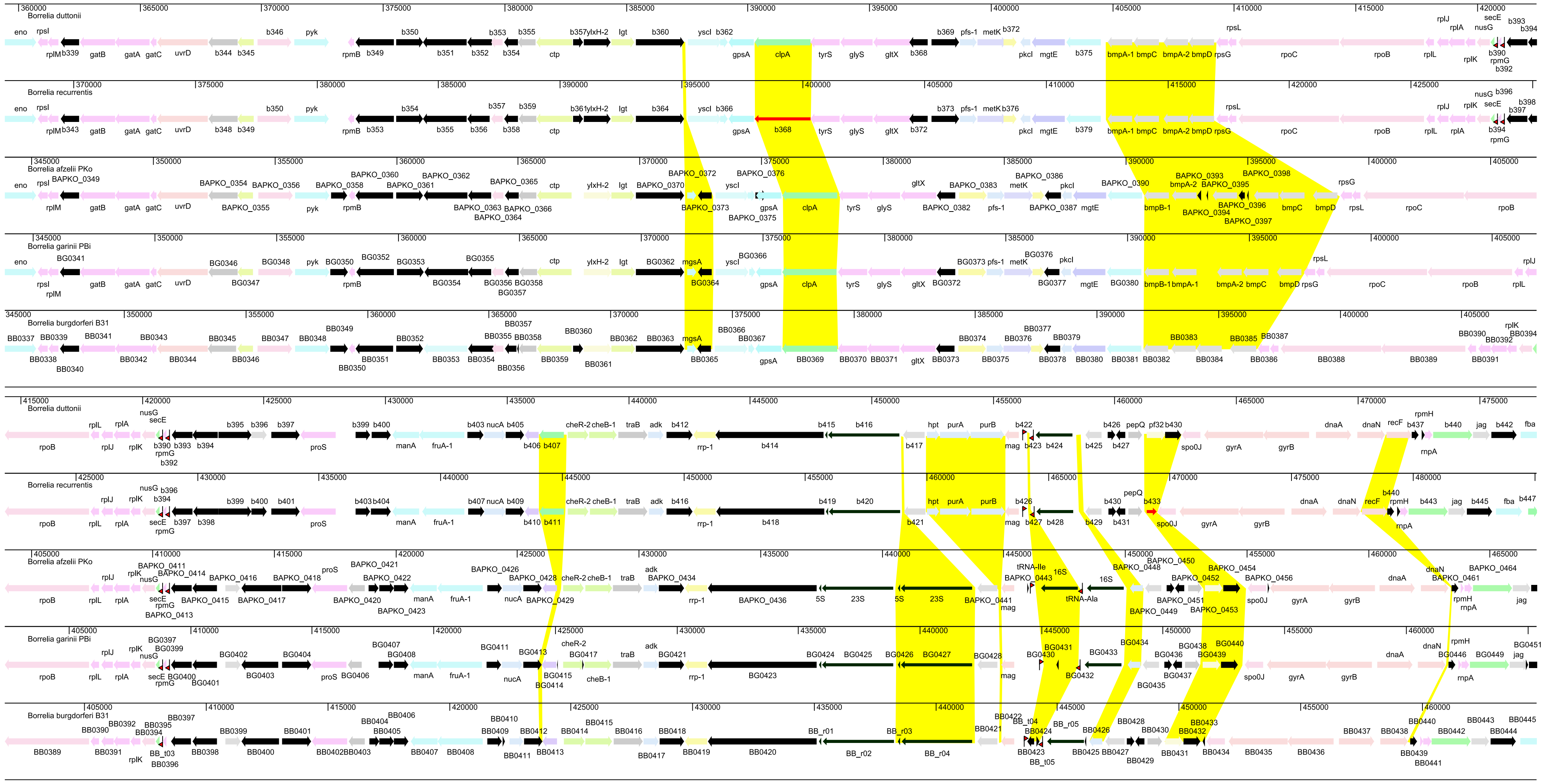
Borrelia duttonii
Borrelia recurrentis
Borrelia afzelii PKo
Borrelia garinii PBi
Borrelia burgdorferi B31
Borrelia duttonii
Borrelia recurrentis
Borrelia afzelii PKo
Borrelia garinii PBi
Borrelia burgdorferi B31

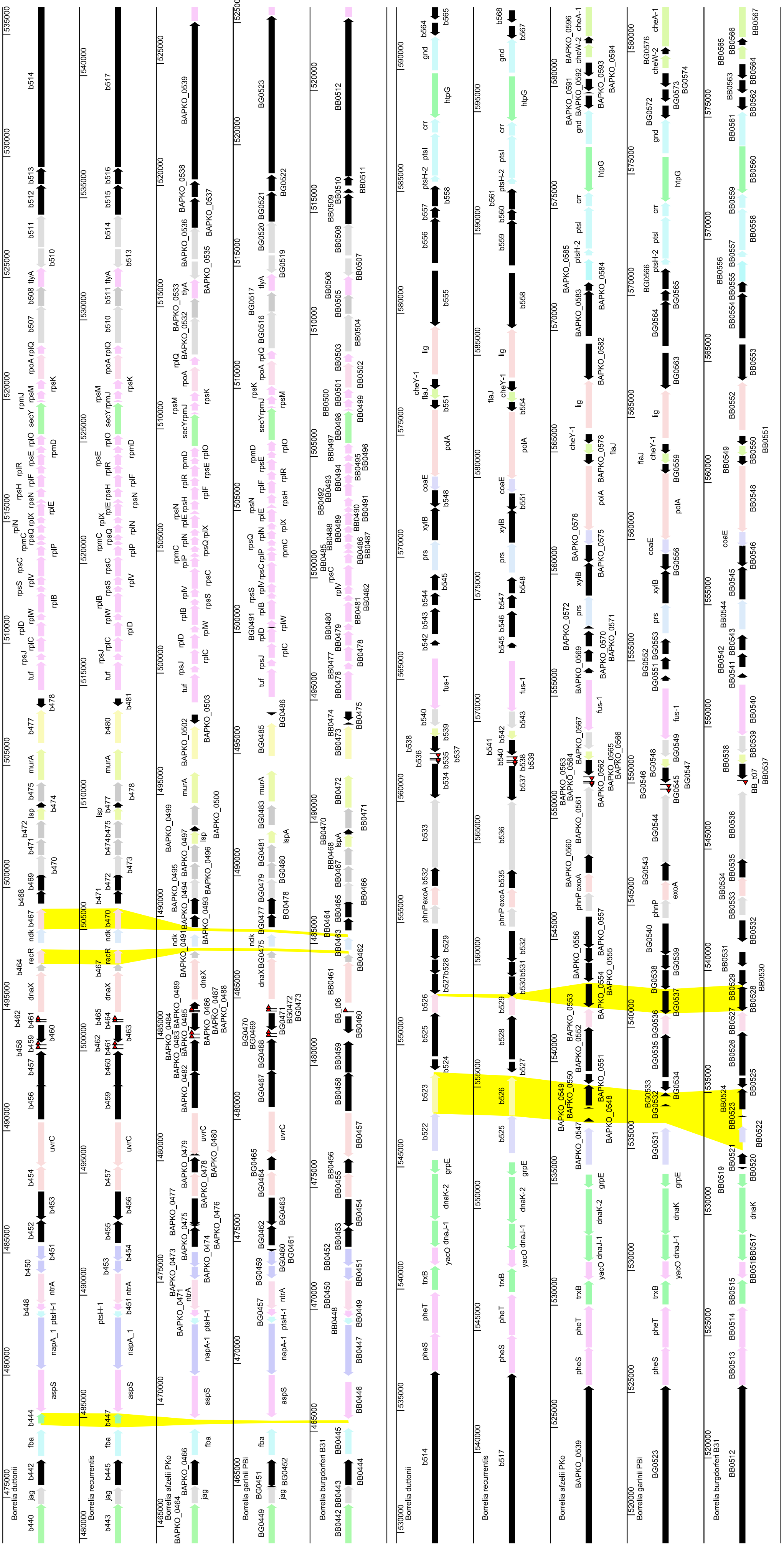
Borrelia duttonii
Borrelia recurrentis
Borrelia afzelii PKo
Borrelia garinii PBi
Borrelia burgdorferi B31

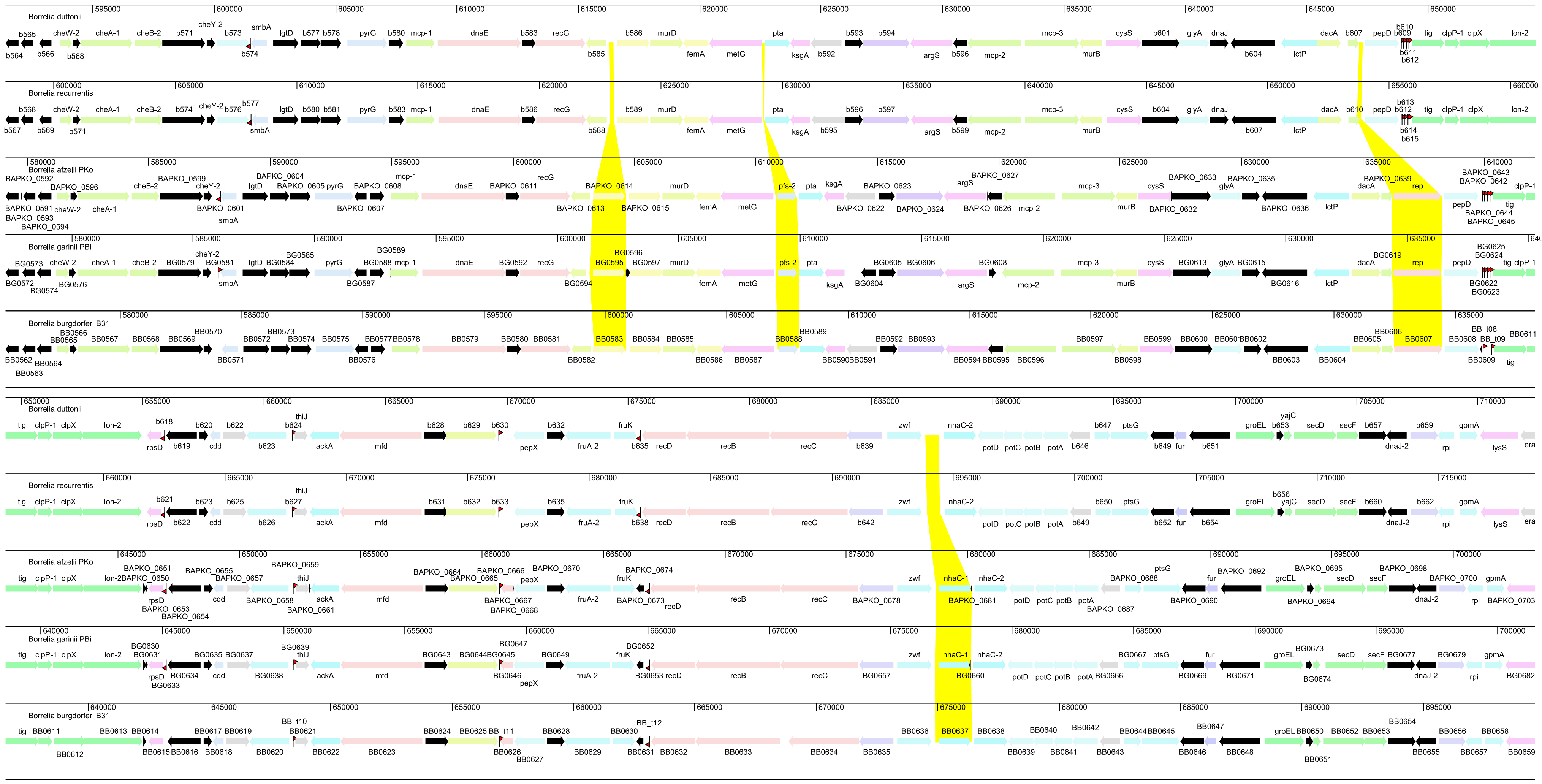
Borrelia duttonii
Borrelia recurrentis
Borrelia afzelii PKo
Borrelia garinii PBi
Borrelia burgdorferi B31
Borrelia duttonii
Borrelia recurrentis
Borrelia afzelii PKo
Borrelia garinii PBi
Borrelia burgdorferi B31

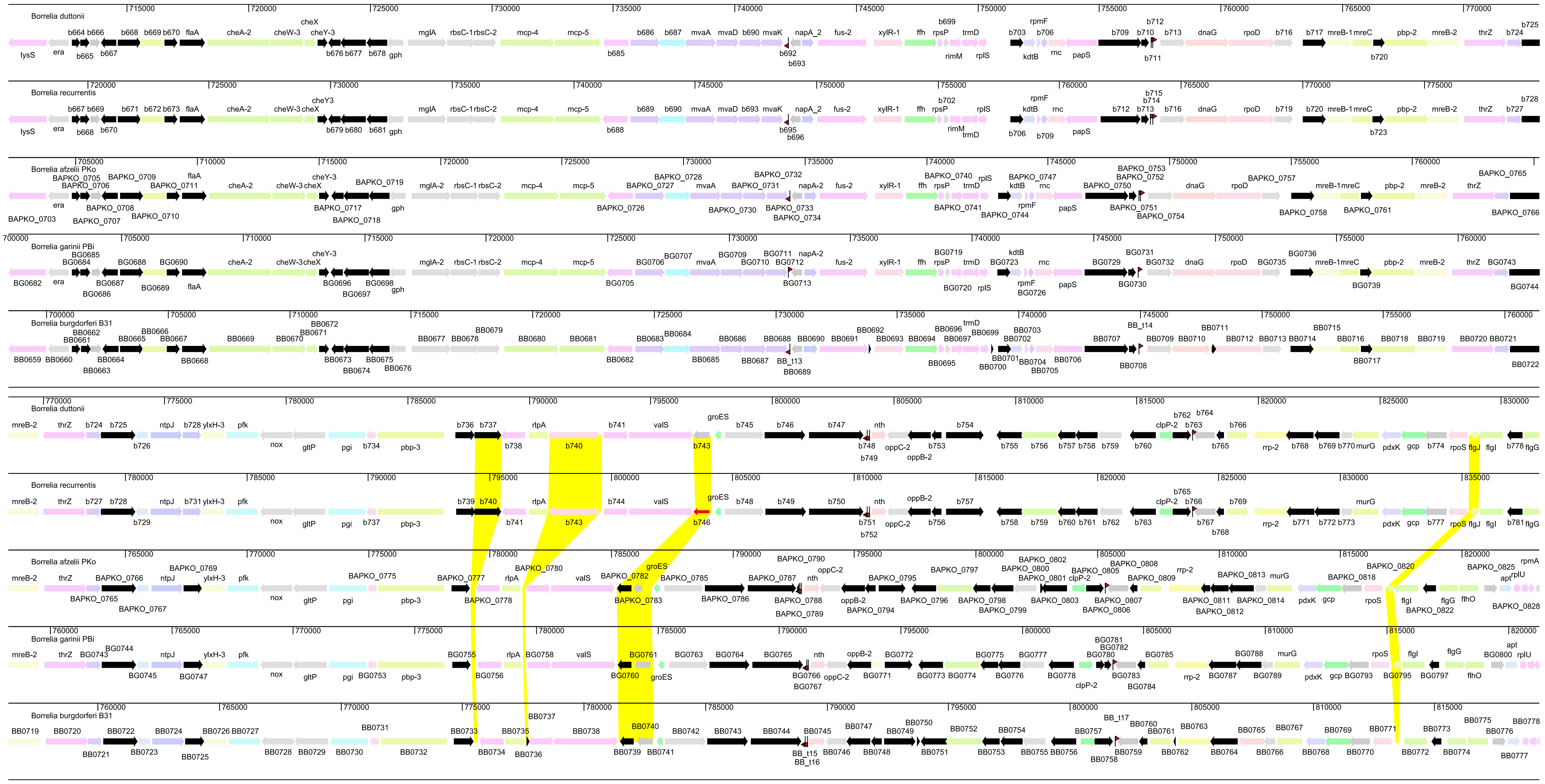
Borrelia duttonii
Borrelia recurrentis
Borrelia afzelii PKo
Borrelia garinii PBi
Borrelia burgdorferi B31

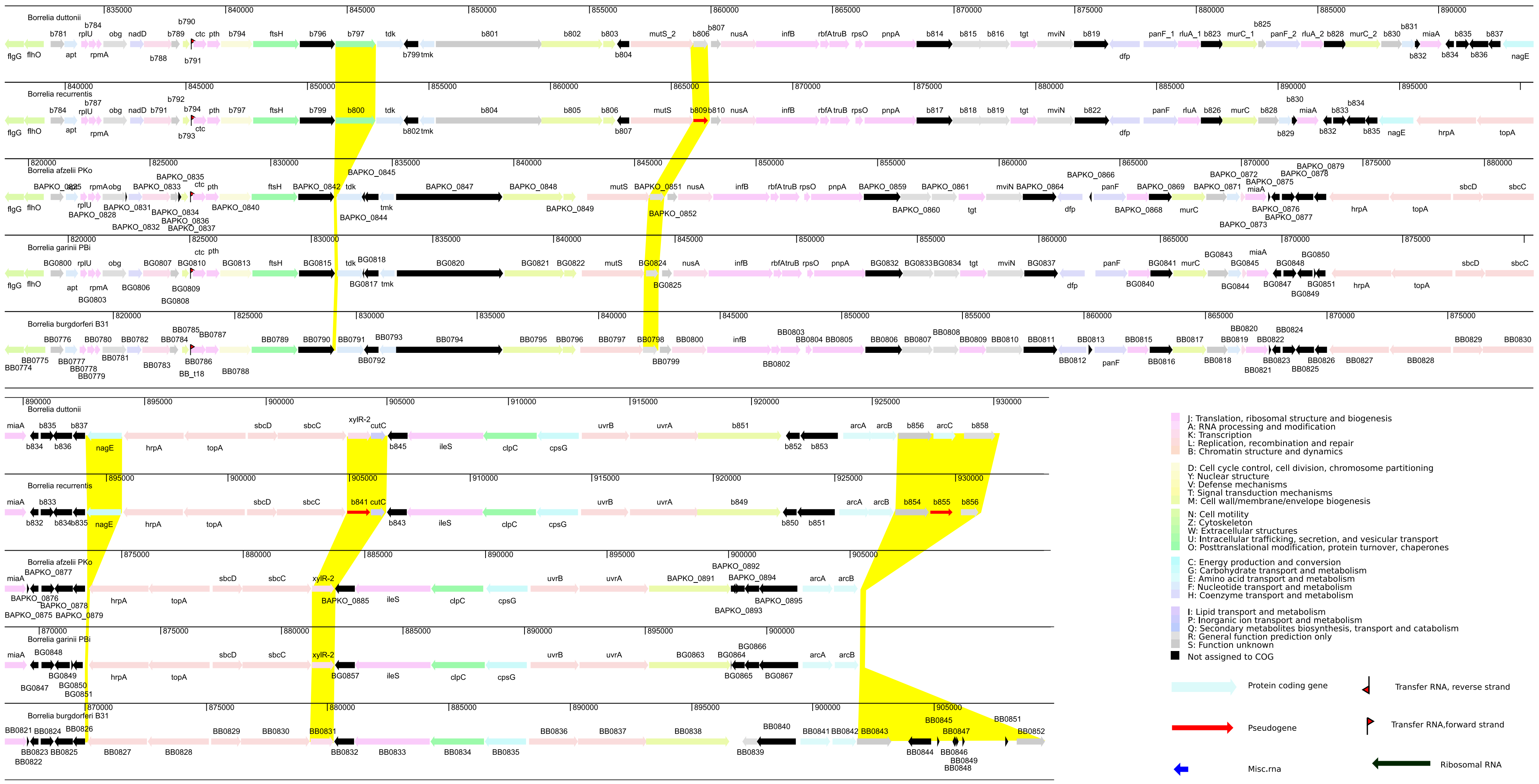

Borrelia duttonii
Borrelia recurrentis
Borrelia afzelii PKo
Borrelia garinii PBi
Borrelia burgdorferi B31
J: Translation, ribosomal structure and biogenesis
A: RNA processing and modification
K: Transcription
L: Replication, recombination and repair
B: Chromatin structure and dynamics
D: Cell cycle control, cell division, chromosome partitioning
Y: Nuclear structure
V: Defense mechanisms
T: Signal transduction mechanisms
M: Cell wall/membrane/envelope biogenesis
N: Cell motility
Z: Cytoskeleton
W: Extracellular structures
U: Intracellular trafficking, secretion, and vesicular transport
O: Posttranslational modification, protein turnover, chaperones
C: Energy production and conversion
G: Carbohydrate transport and metabolism
E: Amino acid transport and metabolism
F: Nucleotide transport and metabolism
H: Coenzyme transport and metabolism
I: Lipid transport and metabolism
P: Inorganic ion transport and metabolism
Q: Secondary metabolites biosynthesis, transport and catabolism
R: General function prediction only
S: Function unknown
Not assigned to COG
Protein coding gene
Transfer RNA, reverse strand
Pseudogene
Transfer RNA,forward strand
Misc.rna
Ribosomal RNA
