# Supplementary material for: The Genome of Borrelia recurrentis, the Agent of Deadly Louse-Borne Relapsing Fever, Is a Degraded Subset of Tick-Borne Borrelia duttonii
Source: PLoS Genet. 2008 Sep 12;4(9):e1000185. doi: 10.1371/journal.pgen.1000185 (PMC2525819; doi:10.1371/journal.pgen.1000185)
Supplement: Figure S2 — B. duttonii and B. recurrentis plasmids. The large B. duttonii-lp165 and B. recurrentis-lp124 plasmids, which demonstrate extensive similarity, are shown side by side, with shaded areas indicating regions of difference. Genes are colored according to their repeat-family membership (Table 2). (1.78 MB PDF) [file pgen.1000185.s002.pdf]

Large plasmids: *Borrelia duttonii*-lp165 and *Borrelia recurrentis*-lp124

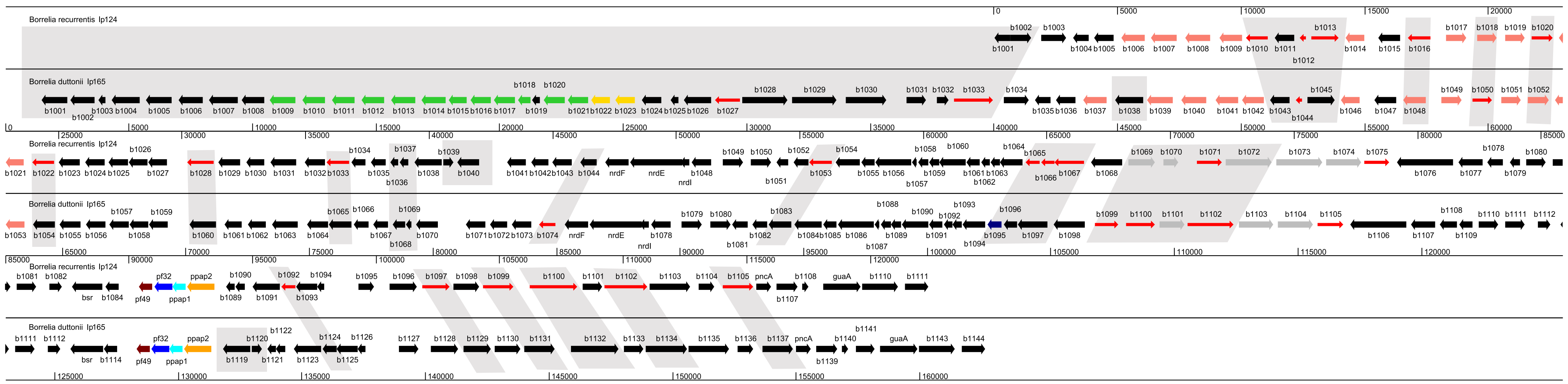

Small plasmids: *Borrelia duttonii*

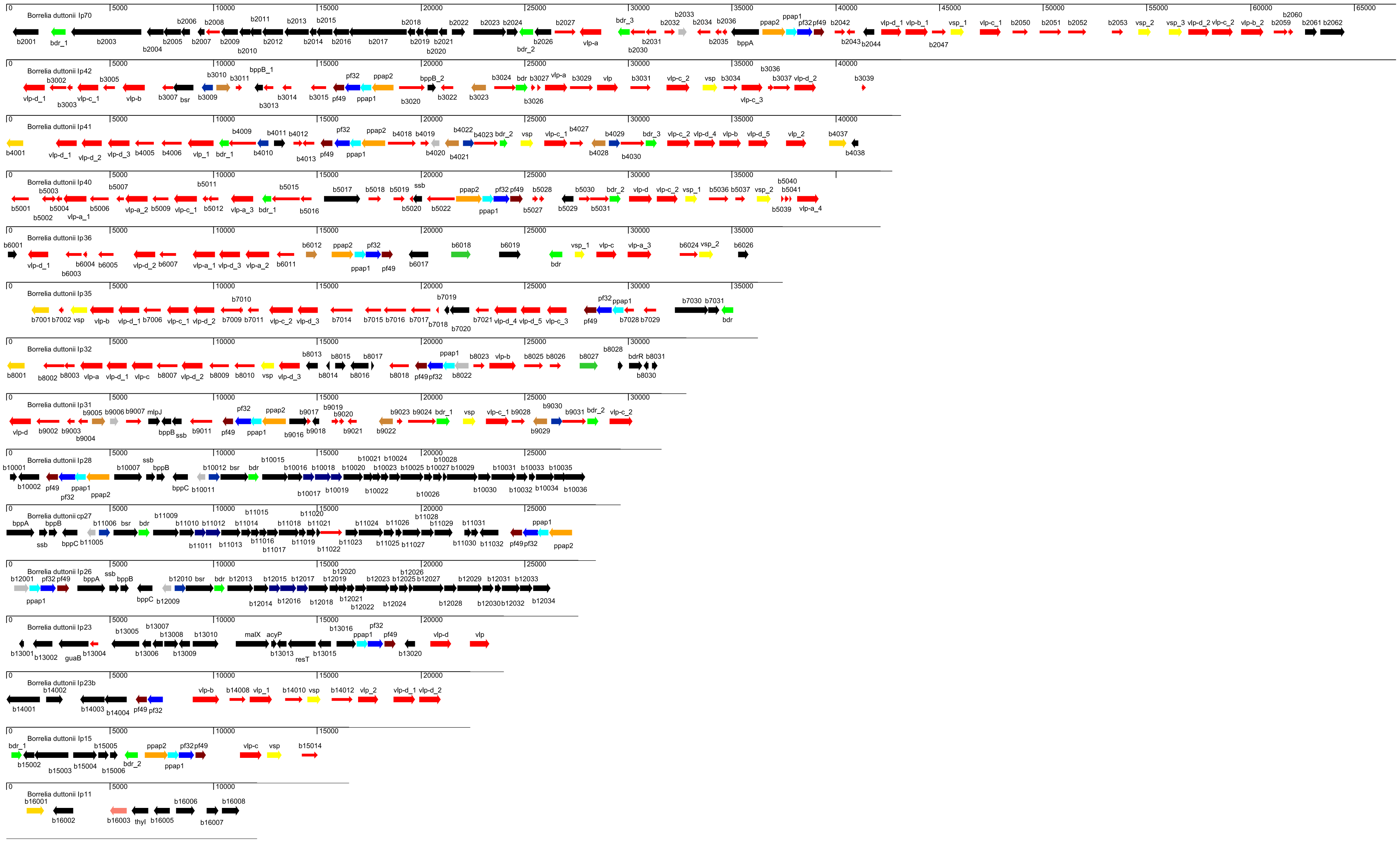

Small plasmids: *Borrelia recurrentis*

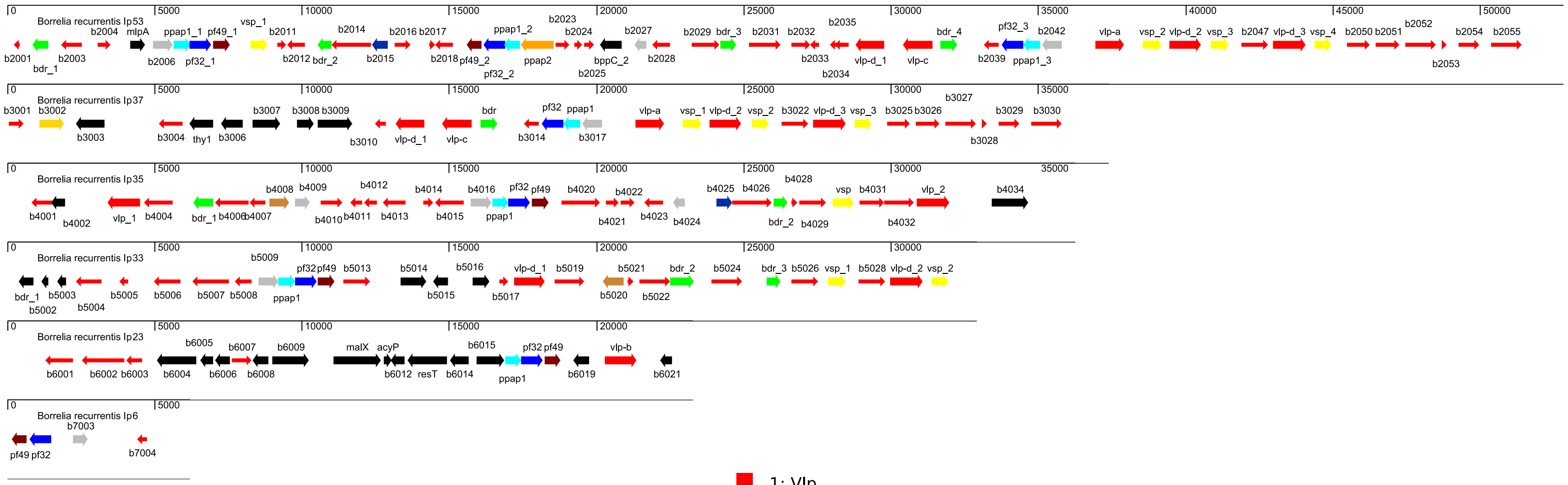

1: Vlp
5: Bdr
2: Plasmid partition pf32
10: Vsp
3: Plasmid partition pf49

4: Plasmid partionning associated protein 1
13: Uncharacterized conserved protein (lipoprotein)
44: Uncharacterized conservedprotein (lipoprotein)
6: Plasmid partionning associated protein 2
14: family 115-like protein
54: ORFk-like protein
55: Antigen P35-like protein
7: Uncharacterized conserved protein

25: Mlpl-likeprotein
76: Uncharacterized conserved protein
84: putative lipoprotein

Protein coding gene

Pseudogene
