# Supplementary figures and images for: The Genome of Borrelia recurrentis, the Agent of Deadly Louse-Borne Relapsing Fever, Is a Degraded Subset of Tick-Borne Borrelia duttonii
Source: PLoS Genet. 2008 Sep 12;4(9):e1000185. doi: 10.1371/journal.pgen.1000185 (PMC2525819; doi:10.1371/journal.pgen.1000185)

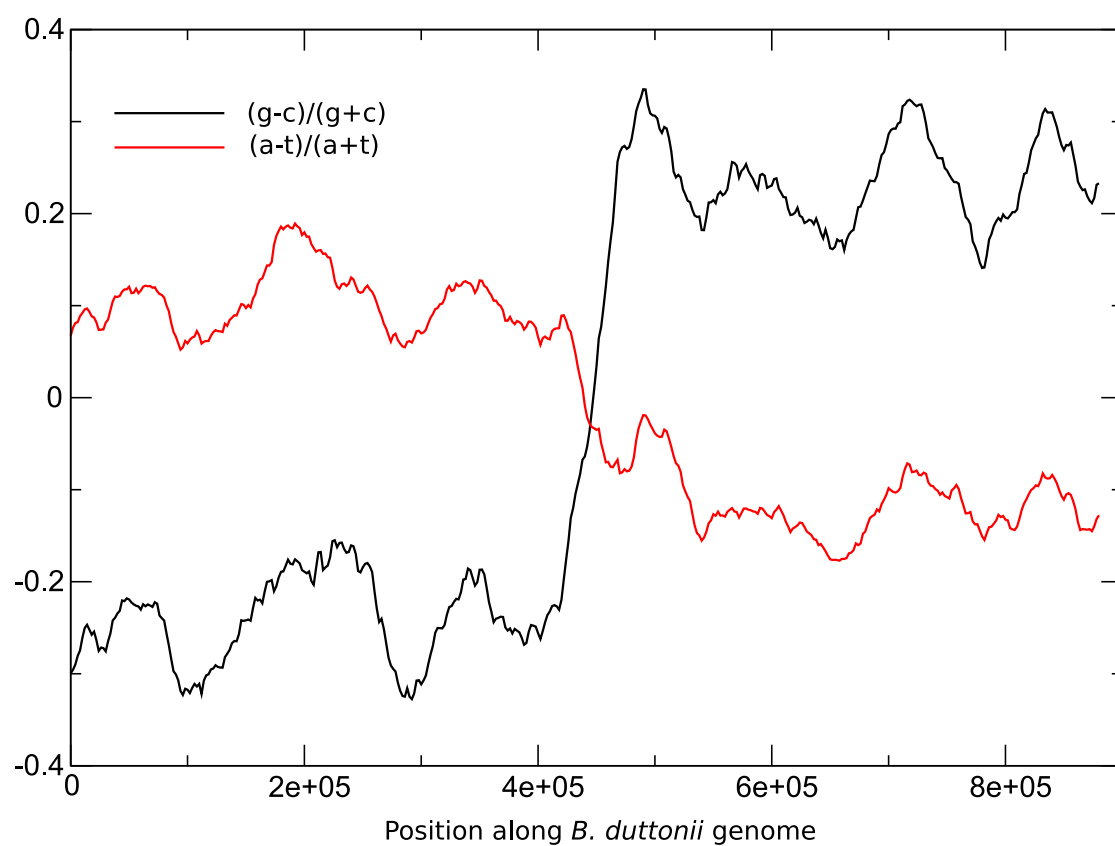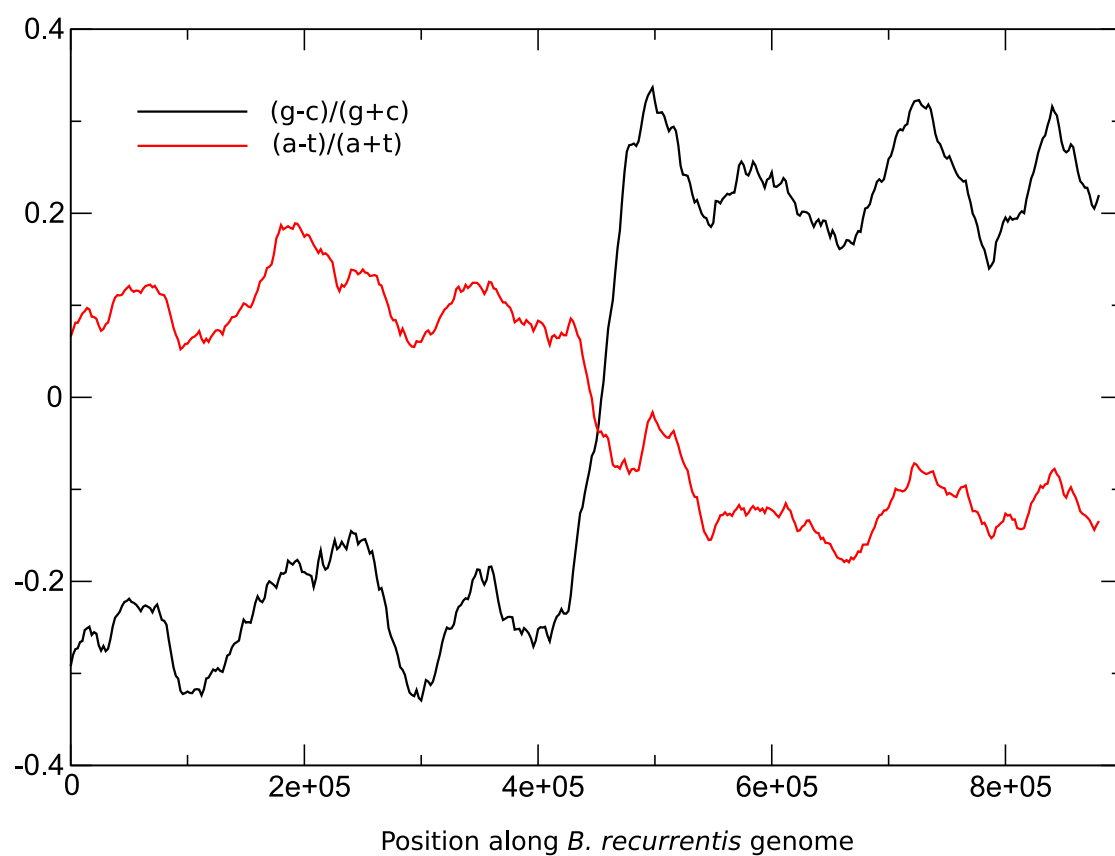

Supplement: Figure S3 — GC and AT skews of B. recurrentis and B. duttonii chromosomes showing reversal near the origin of replication. (0.05 MB PDF) [file pgen.1000185.s003.pdf]

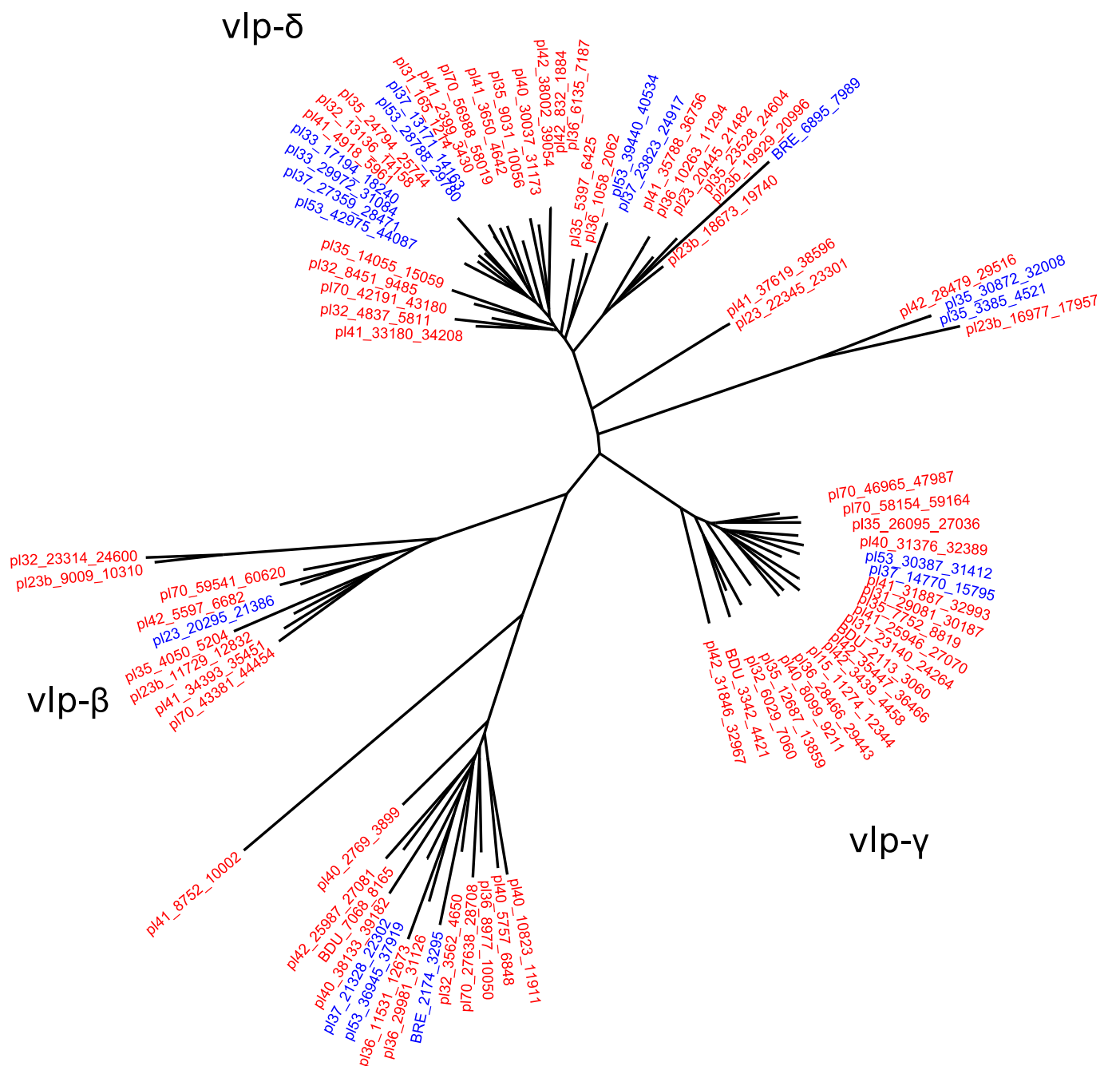

Supplement: Figure S4 — Phylogenetic tree of intact vlp genes in the genomes of B. duttonii (in red) and B. recurrentis (in blue). The genes were aligned with the MUSCLE program [86] and the tree was built using PHYML [89]. (0.40 MB PDF) [file pgen.1000185.s004.pdf]

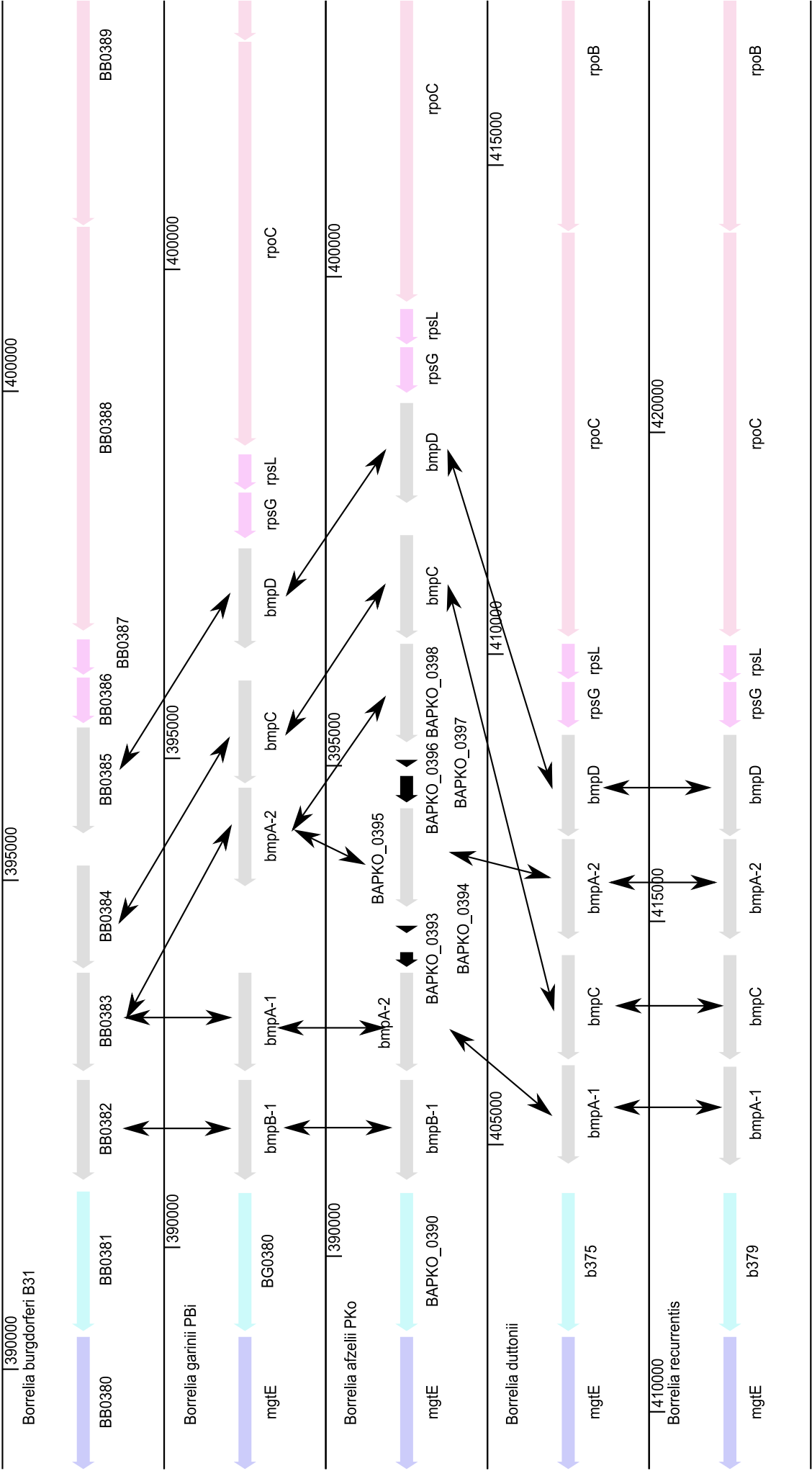

Supplement: Figure S5 — Comparison of the Bmp gene family in five borreliae genomes indicates structural rearrangements in Lyme disease group borreliae. Genes are colored according to predicted functional category (Figure S1). (0.15 MB PDF) [file pgen.1000185.s005.pdf]

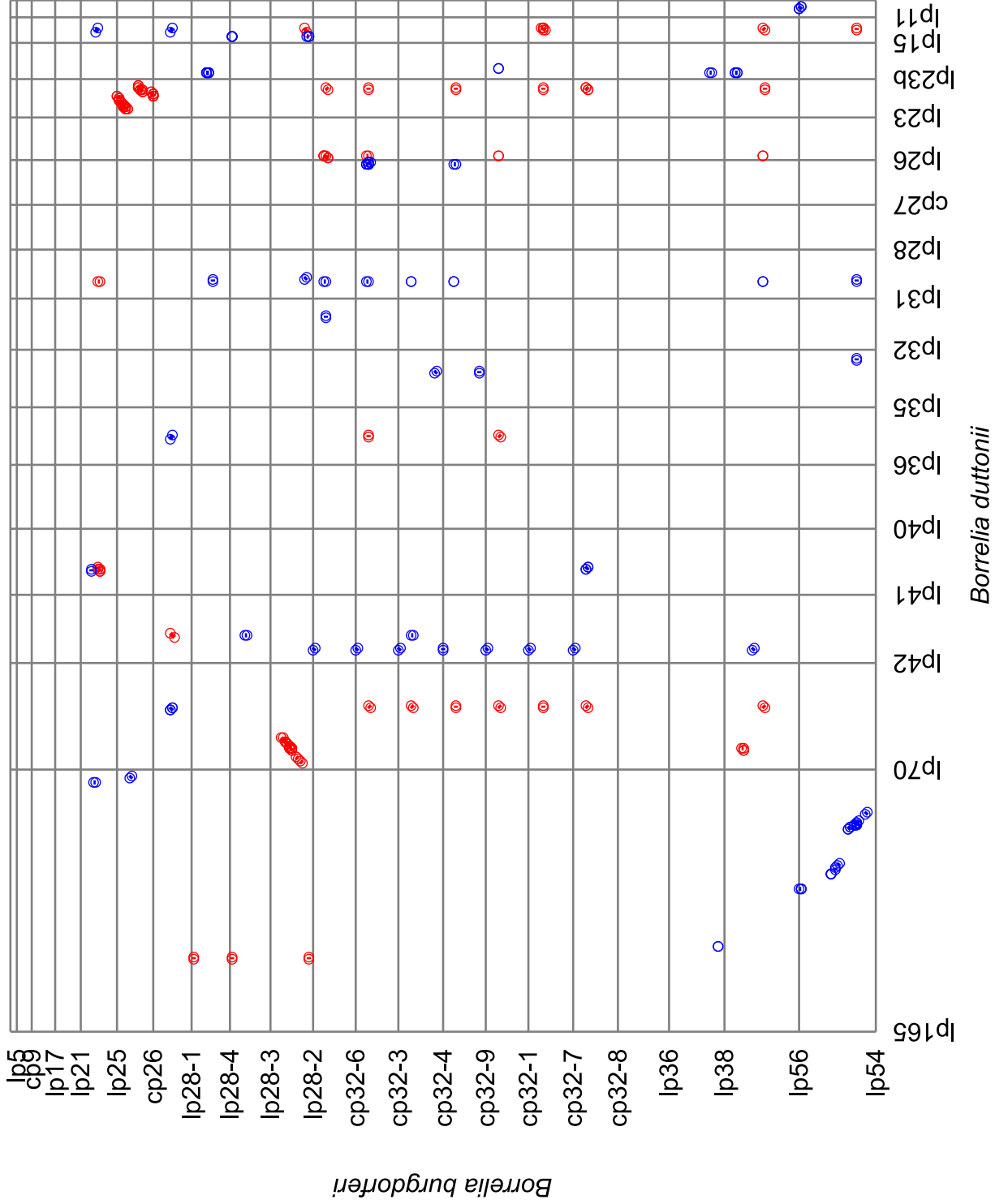

Supplement: Figure S6 — Dot plot showing the extensive similarity between B. duttonii and B. burgdorferi plasmids. This figure was constructed using the PROmer program from the MUMmer package. Red segments correspond to same strand matches, while blue segments correspond to opposite strand matches. (0.07 MB PDF) [file pgen.1000185.s006.pdf]
