# Supplementary material for: The Genome of Borrelia recurrentis, the Agent of Deadly Louse-Borne Relapsing Fever, Is a Degraded Subset of Tick-Borne Borrelia duttonii
Source: PLoS Genet. 2008 Sep 12;4(9):e1000185. doi: 10.1371/journal.pgen.1000185 (PMC2525819; doi:10.1371/journal.pgen.1000185)

First migration

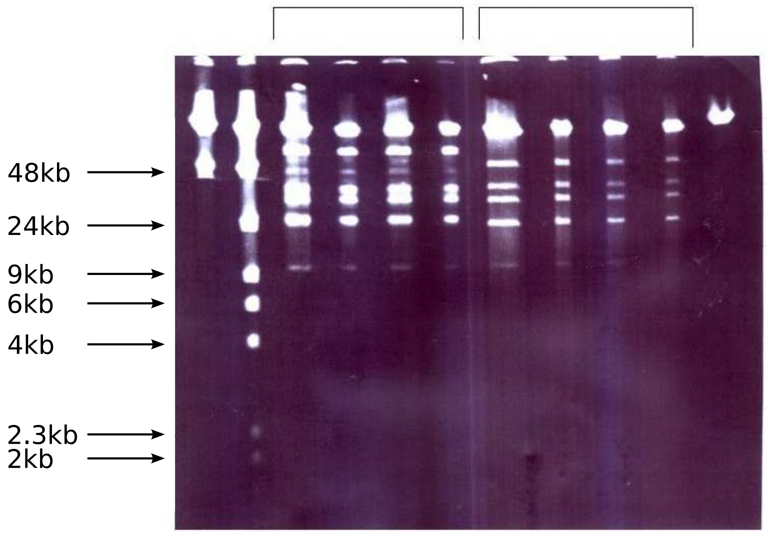

Second migration

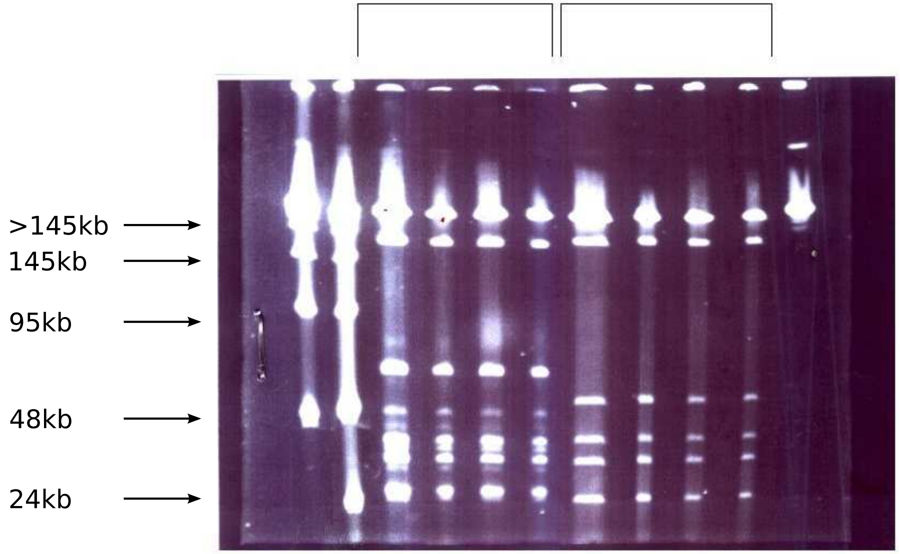

Third migration

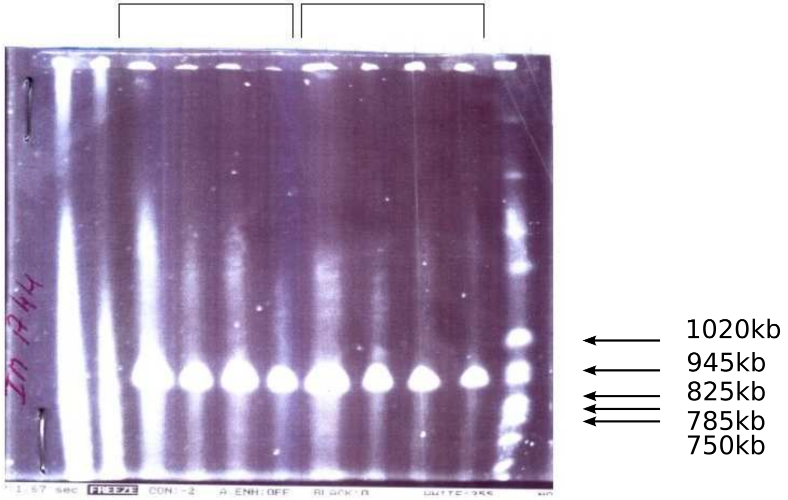

Supplement: Figure S7 — Pulse field gel electrophoresis images of B. duttonii and B. recurrentis. (0.15 MB PDF) [file pgen.1000185.s007.pdf]
