# Supplementary material for: The Genome of Borrelia recurrentis, the Agent of Deadly Louse-Borne Relapsing Fever, Is a Degraded Subset of Tick-Borne Borrelia duttonii
Source: PLoS Genet. 2008 Sep 12;4(9):e1000185. doi: 10.1371/journal.pgen.1000185 (PMC2525819; doi:10.1371/journal.pgen.1000185)
Supplement: Table S1 — List of genes which are either absent, split, or in reduced number in B. recurrentis when compared to B. duttonii. (0.03 MB DOC) [file pgen.1000185.s008.doc]

**Supplementary Table 1**. List of genes which are either absent, split, or in reduced number in *B. recurrentis* when compared to *B. duttonii*.

| Absent |  |  |  |
| --- | --- | --- | --- |
|  | ATPase | Chromosome partitioning | BDU_429 |
|  | Antigen p35-like | fibronectin-binding lipoprotein BBK32 homologue | BDU_1 |
|  | Uncharacterized conserved protein |  | BDU_430 |
| Split |  |  |  |
|  | recA |  | BDU_135 |
|  | recJ^1^ |  | BDU_257 |
|  | Smf |  | BDU_300 |
|  | mutS |  | BDU_101 |
|  | YplQ | Hemolysin III | BDU_120 |
|  | malX |  | BDU_119 |
|  | malQ |  | BDU_165 |
|  | xylR2 | Xylose operon | BDU_843 |
|  | glpK | Glycerol kinase | BDU_241 |
|  | glpA |  | BDU_244 |
|  | bacA^1^ |  | BDU_261 |
|  | oppA-1 |  | BDU_329 |
|  | ATP-dependent Clp protease, subunit A |  | BDU_364 |
|  | Competence protein F |  | BDU_806 |
|  | Hypothetical protein |  | BDU_743 |
| Reduced number |  |  |  |
|  | Vlp | Antigenic lipoprotein |  |
|  | Vsp | Antigenic lipoprotein |  |
|  | Bdr |  |  |
|  | PF32 | Plasmid partition |  |
|  | PF49 | Plasmid partition |  |
|  | PPAP-1 | Plasmid partition |  |
|  | PPP-2 | Plasmid partition |  |
|  | Bsr |  |  |
|  | PTS system component |  |  |
|  | Transposase-like |  |  |
|  | Mlpl-like lipoprotein |  |  |
|  | BppB |  |  |
|  | Family 115-like |  |  |

1-This gene is duplicated, one copy is split.
