# Supplementary material for: The Genome of Borrelia recurrentis, the Agent of Deadly Louse-Borne Relapsing Fever, Is a Degraded Subset of Tick-Borne Borrelia duttonii
Source: PLoS Genet. 2008 Sep 12;4(9):e1000185. doi: 10.1371/journal.pgen.1000185 (PMC2525819; doi:10.1371/journal.pgen.1000185)
Supplement: Table S2 — A. Split and truncated genes on the Borrelia chromosome. B. List of genes unconserved between the five borreliae. (0.08 MB DOC) [file pgen.1000185.s009.doc]

**Supplementary Table 2.** **A**. Split and truncated genes on the *Borrelia* chromosomes. **B**. List of genes unconserved between the five borreliae.

A.

| **BBU** | **BGA** | **BAF** | **BDU** | **BRE** |
| --- | --- | --- | --- | --- |
| BB0021 S-adenosylmethionine: tRNA ribosyltransferase-isomerase  BB0223/24 lipoprotein  BB0356/57 hypothetical protein  BB0410/11 nucA endonuclease precursor  BB0510/11 putative hydrolase  BB0521/22 NH(3)-dependent NAD+ synthetase  BB0710/11 DNA primase (dnaG) | BB0078/79 lipoprotein releasing system, permease proteinBG0086/87 magnesium chelatase family proteinBG0125/126 BG0226/27 lipoprotein  BG0486 lipoprotein  BG0604 competence locus E, putative  BG0780/81 lipoprotein  BG0533 inositol monophosphatase | BAPKO_0075 peptide chain release factor 2 (prfB)BAPKO_0125/126BAPKO_0231/32 lipoproteinBAPKO_0190/191 BAPKO_0206/207 hemolysin  BAPKO_0311/312 cell division protein BAPKO_0783 hypothetical protein | BDU_4, vlp pseudogene  BDU_6. vlp pseudogene | BRE_1, vlp pseudogene  BRE_2, vlp pseudogene  BRE_5, vlp pseudogene  BRE_100, mutS  BRE_118, malX, PTS system, maltose and glucose-specific IIBC component  BRE_119, ylpQ, hemolysin III  BRE_134, recA  BRE_164, malQ, 4-alpha-glucanotransferase  BRE_240, glpK, glycerol kinase  BRE_243, glpA  BRE_260, bacA  BRE_261, recJ  BRE_304, smf  BRE_333, oppA-1  BRE_368, clpA, ATP-dependent Clp protease, subunit A  BRE_433, uncharacterized conserved protein  BRE_746, uncharacterised conserved protein pseudogene  BRE_809, competence protein F  BRE_841, xylR-2, xylose operon regulatory protein  BRE_855 arcC |

**B.**

| **BBU** | **BGA** | **BAF** | **BDU** | **BRE** |
| --- | --- | --- | --- | --- |
| BB0024 putative L-sorbosone dehydrogenase | BG0024 putative L-sorbosone dehydrogenase | BAPKO_0023 | - | - |
| - | - | - | BDU_127 maf protein | BRE_126 maf protein |
| BB0158 antigen, S2, putative | BG0156 antigen, S2, putative | BAPKO_0159 antigen, S2, putative | - | - |
| BB0159 antigen, S2, putative | BG0157 antigen, S2, putative | BAPKO_0160 antigen, S2, putative | - | - |
| - | - | - | BDU_241 glpT | BRE_240 glpT |
| - | - | - | BDU_243 glpQ | BRE_242 glpQ |
| - | - | - | BDU_313 RecN | BRE_317 RecN |
| BB0330 oligopeptide ABC transporter (oppA-3) | BG0331 oligopeptide ABC transporter (oppA-3) | BAPKO_0339 oligopeptide ABC transporter (oppA-3) | - | - |
| BB0364 methylglyoxal synthase | BG0363 methylglyoxal synthase | BAPKO_0372 methylglyoxal synthase | - | - |
| BB0365 lipoprotein LA7 | BG0364 lipoprotein LA7 | BAPKO_0373 lipoprotein LA7 | - | - |
| BB0382 basic membrane protein B (bmpB-1) | BG0381 basic membrane protein B (bmpB-1) | BAPKO_0391 basic membrane protein B (bmpB-1) | - | - |
| - | - | BAPKO_0396 partial bmpC | - | - |
| - | BG0384 bmpA-2 | BAPKO_0392 bmpA-2 | BDU_378 bmpA-2 | BRE_382 bmpA-2 |
| - | - | BAPKO_0398 bmpA-3 | - | - |
| - | - | - | BDU_407 peptidyl-prolyl cis-trans isomerase | BRE_411 peptidyl-prolyl cis-trans isomerase |
| - | - | - | BDU_418 hypoxanthine phosphoribosyltransferase (hpt) | BRE_422 hypoxanthine phosphoribosyltransferase (hpt) |
| - | - | - | BDU_419 adenylosuccinate synthase (purA) | BRE_423 adenylosuccinate synthase (purA) |
| - | - | - | BDU_420 adenylosuccinate lyase (purB) | BRE_424 adenylosuccinate lyase (purB) |
| BB0431 Soj gene homologue | BG0439 Soj gene homologue | BAPKO_0453 Soj gene homologue | BDU_429 Soj gene homologue | - |
| - | - | - | BDU_436 recF | BRE_439 recF |
| - | - | - | BDU_444 hsp20 heat shock protein | BRE_447 hsp20 heat shock protein |
| - | - | - | BDU_465 recR | BRE_468 recR |
| - | - | - | BDU_467 putative adenine-specific DNA methyltransferase | BRE_470 putative adenine-specific DNA methyltransferase |
| - | - | - | BDU_523 partially similar to a response regulator receiver (CheY) modulated serine phosphatase | BRE_526 partially similar to a response regulator receiver (CheY) modulated serine phosphatase |
| BB0524 inositol monophosphatase | BG0533 inositol monophosphatase | BAPKO_0550 inositol monophosphatase | - | - |
| BB0528 aldose reductase | BG0537 aldose reductase | BAPKO_0554 aldose reductase | - | - |
| BB0583 hypothetical protein, MATE efflux family protein | BG0595 hypothetical protein, MATE efflux family protein | BAPKO_0614 hypothetical protein, MATE efflux family protein | - | - |
| BB0588 pfs protein (pfs-2) | BG0601 pfs protein (pfs-2) | BAPKO_0619 pfs protein (pfs-2) | - | - |
| BB0607 rep helicase, single-stranded DNA-dependent ATPase | BG0620 rep helicase, single-stranded DNA-dependent ATPase | BAPKO_0640 rep helicase, single-stranded DNA-dependent ATPase | - | - |
| BB0626 small primase-like protein | BG0646 small primase-like protein | BAPKO_0667 small primase-like protein | - | - |
| BB0637 Na+/H+ antiporter (nhaC-1) | BG0659 Na+/H+ antiporter (nhaC-1) | BAPKO_0680 Na+/H+ antiporter (nhaC-1) | - | - |
| - | - | - | BDU_737 similar to a TPR domain protein | BRE_740 similar to a TPR domain protein |
| - | - | - | BDU_740 ATP-dependent helicase, DinG family | BRE_743 ATP-dependent helicase, DinG family |
| - | BG0795 hypothetical protein | BAPKO_0820 hypothetical protein | BDU_776 flagellum-specific muramidase (flgJ) | BRE_779 flagellum-specific muramidase (flgJ) |
| - | - | - | BDU_797 trypsin-like serine proteases | BRE_800 trypsin-like serine proteases |
| - | - | - | BDU_804 hypothetical protein | BRE_807 hypothetical protein |
| - | - | - | BDU_828 uncharacterized conserved protein (due to duplication of panF locus) | - |
| - | - | - | BDU_838 nagE, Sugar Specific PTS family | BRE_836 nagE, Sugar Specific PTS family |
| - | - | - | BDU_844 copper homeostasis protein cutC | BRE_842 copper homeostasis protein cutC |
| - | - | - | BDU_857, arcC | BRE_855 arcC pseudogene |
