# Supplementary material for: The Genome of Borrelia recurrentis, the Agent of Deadly Louse-Borne Relapsing Fever, Is a Degraded Subset of Tick-Borne Borrelia duttonii
Source: PLoS Genet. 2008 Sep 12;4(9):e1000185. doi: 10.1371/journal.pgen.1000185 (PMC2525819; doi:10.1371/journal.pgen.1000185)
Supplement: Table S3 — List of the different variable large proteins in the B. duttonii and B. recurrentis genomes. A. B. recurrentis; B. B. duttonii; C. Repartition of the Vlp genes among different classes in the two borreliae. (0.15 MB DOC) [file pgen.1000185.s010.doc]

**Supplementary Table 3.** List of the different variable large proteins in the *B. duttonii* and *B. recurrentis* genomes. **A**. *B. recurrentis*; **B**. *B. duttonii*; **C**. Repartition of the Vlp genes among different classes in the two borreliae.

A.

| **Plasmid** | **Positions** | **locus_tag** | **vlp class** | **Annotation** | **Putative RBS** | **RBS position** |
| --- | --- | --- | --- | --- | --- | --- |
| lp23 | 20295-21386 | BRE_6020 | vlp-b | vlp gene, beta subfamily |  |  |
| lp33 | 17194-18240 | BRE_5018 | vlp-d | vlp gene, delta subfamily | GGAGG | 17181 |
| lp33 | 29972-31084 | BRE_5029 | vlp-d | vlp gene, delta subfamily | GGAGG | 29968 |
| lp35 | 3385-4521 | BRE_4003 | vlp | vlp gene |  |  |
| lp35 | 30872-32008 | BRE_4033 | vlp | vlp gene |  |  |
| lp37 | 13171-14163 | BRE_3011 | vlp-d | vlp gene, delta subfamily |  |  |
| lp37 | 14770-15795 | BRE_3012 | vlp-c | vlp gene, gamma subfamily |  |  |
| lp37 | 21328-22302 | BRE_3018 | vlp-a | vlp gene, alpha subfamily |  |  |
| lp37 | 23823-24917 | BRE_3020 | vlp-d | vlp gene, delta subfamily | GGAGG | 23807 |
| lp37 | 27359-28471 | BRE_3023 | vlp-d | vlp gene, delta subfamily | GGAGG | 27355 |
| lp53 | 28788-29780 | BRE_2036 | vlp-d | vlp gene, delta subfamily |  |  |
| lp53 | 30387-31412 | BRE_2037 | vlp-c | vlp gene, gamma subfamily |  |  |
| lp53 | 36945-37919 | BRE_2043 | vlp-a | vlp gene, alpha subfamily |  |  |
| lp53 | 39440-40534 | BRE_2045 | vlp-d | vlp gene, delta subfamily | GGAGG | 39424 |
| lp53 | 42975-44087 | BRE_2048 | vlp-d | vlp gene, delta subfamily | GGAGG | 42971 |
| Chromosome | 2174-3295 | BRE_3 | vlp-a | vlp gene, alpha subfamily |  |  |
| Chromosome | 6895-7989 | BRE_4 | vlp-d | vlp gene, delta subfamily |  |  |

B.

| **Plasmid** | **Positions** | **locus_tag** | **vlp class** | **Annotation** | **Putative RBS** | **RBS position** |
| --- | --- | --- | --- | --- | --- | --- |
| lp15 | 11274-12344 | BDU_15012 | vlp-c | vlp gene, gamma subfamily | GGAGG | 11261 |
| lp23 | 20445-21482 | BDU_13021 | vlp-d | vlp gene, delta subfamily | GGAGG | 20432 |
| lp23 | 22345-23301 | BDU_13022 | vlp | vlp gene |  |  |
| lp31 | 165-1214 | BDU_9001 | vlp-d | vlp gene, delta subfamily |  |  |
| lp31 | 23140-24264 | BDU_9027 | vlp-c | vlp gene, gamma subfamily |  |  |
| lp31 | 29081-30187 | BDU_9033 | vlp-c | vlp gene, gamma subfamily | GGAGG | 29068 |
| lp32 | 3562-4650 | BDU_8004 | vlp-a | vlp gene, alpha subfamily |  |  |
| lp32 | 4837-5811 | BDU_8005 | vlp-d | vlp gene, delta subfamily |  |  |
| lp32 | 6029-7060 | BDU_8006 | vlp-c | vlp gene, gamma subfamily |  |  |
| lp32 | 8451-9485 | BDU_8008 | vlp-d | vlp gene, delta subfamily |  |  |
| lp32 | 13136-14158 | BDU_8012 | vlp-d | vlp gene, delta subfamily |  |  |
| lp32 | 23314-24600 | BDU_8024 | vlp-b | vlp gene, beta subfamily | GGAGG | 23304 |
| lp35 | 4050-5204 | BDU_7004 | vlp-b | vlp gene, beta subfamily |  |  |
| lp35 | 5397-6425 | BDU_7005 | vlp-d | vlp gene, delta subfamily |  |  |
| lp35 | 7752-8819 | BDU_7007 | vlp-c | vlp gene, gamma subfamily |  |  |
| lp35 | 9031-10056 | BDU_7008 | vlp-d | vlp gene, delta subfamily |  |  |
| lp35 | 12687-13859 | BDU_7012 | vlp-c | vlp gene, gamma subfamily |  |  |
| lp35 | 14055-15059 | BDU_7013 | vlp-d | vlp gene, delta subfamily |  |  |
| lp35 | 23528-24604 | BDU_7022 | vlp-d | vlp gene, delta subfamily |  |  |
| lp35 | 24794-25744 | BDU_7023 | vlp-d | vlp gene, delta subfamily |  |  |
| lp35 | 26095-27036 | BDU_7024 | vlp-c | vlp gene, gamma subfamily |  |  |
| lp36 | 1058-2062 | BDU_6002 | vlp-d | vlp gene, delta subfamily |  |  |
| lp36 | 6135-7187 | BDU_6006 | vlp-d | vlp gene, delta subfamily |  |  |
| lp36 | 8977-10050 | BDU_6008 | vlp-a | vlp gene, alpha subfamily |  |  |
| lp36 | 10263-11294 | BDU_6009 | vlp-d | vlp gene, delta subfamily |  |  |
| lp36 | 11531-12673 | BDU_6010 | vlp-a | vlp gene, alpha subfamily |  |  |
| lp36 | 28466-29443 | BDU_6022 | vlp-c | vlp gene, gamma subfamily | GGAGG | 28436 |
| lp36 | 29981-31126 | BDU_6023 | vlp-a | vlp gene, alpha subfamily | GGAGG | 29968 |
| lp40 | 2769-3899 | BDU_5005 | vlp-a | vlp gene, alpha subfamily |  |  |
| lp40 | 5757-6848 | BDU_5008 | vlp-a | vlp gene, alpha subfamily |  |  |
| lp40 | 8099-9211 | BDU_5010 | vlp-c | vlp gene, gamma subfamily |  |  |
| lp40 | 10823-11911 | BDU_5013 | vlp-a | vlp gene, alpha subfamily |  |  |
| lp40 | 30037-31173 | BDU_5033 | vlp-d | vlp gene, delta subfamily |  |  |
| lp40 | 31376-32389 | BDU_5034 | vlp-c | vlp gene, gamma subfamily | GGAGG | 31363 |
| lp40 | 38133-39182 | BDU_5042 | vlp-a | vlp gene, alpha subfamily |  |  |
| lp41 | 2399-3430 | BDU_4002 | vlp-d | vlp gene, delta subfamily |  |  |
| lp41 | 3650-4642 | BDU_4003 | vlp-d | vlp gene, delta subfamily |  |  |
| lp41 | 4918-5961 | BDU_4004 | vlp-d | vlp gene, delta subfamily |  |  |
| lp41 | 8752-10002 | BDU_4007 | vlp | vlp gene |  |  |
| lp41 | 25946-27070 | BDU_4026 | vlp-c | vlp gene, gamma subfamily | GGAGG | 25933 |
| lp41 | 31887-32993 | BDU_4032 | vlp-c | vlp gene, gamma subfamily | GGAGG | 31874 |
| lp41 | 33180-34208 | BDU_4033 | vlp-d | vlp gene, delta subfamily |  |  |
| lp41 | 34393-35451 | BDU_4034 | vlp-b | vlp gene, beta subfamily |  |  |
| lp41 | 35788-36756 | BDU_4035 | vlp-d | vlp gene, delta subfamily |  |  |
| lp41 | 37619-38596 | BDU_4036 | vlp | vlp gene |  |  |
| lp42 | 832-1884 | BDU_3001 | vlp-d | vlp gene, delta subfamily |  |  |
| lp42 | 3439-4458 | BDU_3004 | vlp-c | vlp gene, gamma subfamily |  |  |
| lp42 | 5597-6682 | BDU_3006 | vlp-b | vlp gene, beta subfamily |  |  |
| lp42 | 25987-27078 | BDU_3028 | vlp-a | vlp gene, alpha subfamily | GGAGG | 25974 |
| lp42 | 28479-29516 | BDU_3030 | vlp | vlp gene | GGAGG | 28466 |
| lp42 | 31846-32967 | BDU_3032 | vlp-c | vlp gene, gamma subfamily | GGAGG | 31833 |
| lp42 | 35447-36466 | BDU_3035 | vlp-c | vlp gene, gamma subfamily |  |  |
| lp42 | 38002-39054 | BDU_3038 | vlp-d | vlp gene, delta subfamily | GGAGG | 37989 |
| lp70 | 27638-28708 | BDU_2028 | vlp-a | vlp gene, alpha subfamily |  |  |
| lp70 | 42191-43180 | BDU_2045 | vlp-d | vlp gene, delta subfamily |  |  |
| lp70 | 43381-44454 | BDU_2046 | vlp-b | vlp gene, beta subfamily | GGAGG | 43368 |
| lp70 | 46965-47987 | BDU_2049 | vlp-c | vlp gene, gamma subfamily |  |  |
| lp70 | 56988-58019 | BDU_2056 | vlp-d | vlp gene, delta subfamily | GGAGG | 56975 |
| lp70 | 58154-59164 | BDU_2057 | vlp-c | vlp gene, gamma subfamily |  |  |
| lp70 | 59541-60620 | BDU_2058 | vlp-b | vlp gene, beta subfamily | GGAGG | 59528 |
| lp23b | 9009-10310 | BDU_14007 | vlp-b | vlp gene, beta subfamily | GGAGG | 8999 |
| lp23b | 11729-12832 | BDU_14009 | vlp | vlp gene | GGAGG | 11716 |
| lp23b | 16977-17957 | BDU_14013 | vlp | vlp gene |  |  |
| lp23b | 18673-19740 | BDU_14014 | vlp-d | vlp gene, delta subfamily |  |  |
| lp23b | 19929-20996 | BDU_14015 | vlp-d | vlp gene, delta subfamily | GGAGG | 19916 |
| Chromosome | 2113-3060 | BDU_2 | vlp-c | vlp gene, gamma subfamily |  |  |
| Chromosome | 3342-4421 | BDU_3 | vlp-c | vlp gene, gamma subfamily |  |  |
| Chromosome | 7068-8165 | BDU_5 | vlp-a | vlp gene, alpha subfamily |  |  |

C.

|  | Vlp-α | Vlp-β | Vlp-γ | Vlp-δ | Others | Total |
| --- | --- | --- | --- | --- | --- | --- |
| *B. duttonii* | 11 | 7 | 19 | 25 | 6 | 68 |
| *B. recurrentis* | 3 | 1 | 2 | 9 | 2 | 17 |
