# Supplementary material for: The Genome of Borrelia recurrentis, the Agent of Deadly Louse-Borne Relapsing Fever, Is a Degraded Subset of Tick-Borne Borrelia duttonii
Source: PLoS Genet. 2008 Sep 12;4(9):e1000185. doi: 10.1371/journal.pgen.1000185 (PMC2525819; doi:10.1371/journal.pgen.1000185)
Supplement: Table S4 — Parameters of the codon models used in this study. (0.02 MB DOC) [file pgen.1000185.s011.doc]

**Supplementary Table 4**. Parameters of the codon models used in this study

|  | Log likelihood | Number of parameters | ω ratio values | K values |
| --- | --- | --- | --- | --- |
| Model 1 | -1,230,740 | 10 | ω_Bre-Bdu_ = 0.14  ω_0_ = 0.06 | Ka*_Bre_*=0.0016 Ks*_Bre_*=0.0115  Ka*_Bdu_*=0.0009 Ks*_Bdu_*=0.0064 |
| Model 2 | -1,230,737 | 11 | ω_Bre_ = 0.18  ω_Bdu_ = 0.10  ω_0_ = 0.06 | Ka_Bre_=0.0017 Ks_Bre_=0.0092  Ka_Bdu_=0.0009 Ks_Bdu_=0.0087 |
